# General practitioners and emergency departments (GPED)—efficient models of care: a mixed-methods study protocol

**DOI:** 10.1136/bmjopen-2018-024012

**Published:** 2018-10-03

**Authors:** Katherine Morton, Sarah Voss, Joy Adamson, Helen Baxter, Karen Bloor, Janet Brandling, Sean Cowlishaw, Tim Doran, Andrew Gibson, Nils Gutacker, Dan Liu, Sarah Purdy, Paul Roy, Christopher Salisbury, Arabella Scantlebury, Anu Vaittinen, Rose Watson, Jonathan Richard Benger

**Affiliations:** 1 Faculty of Health and Life Sciences, University of the West of England, Bristol, UK; 2 Institute of Health and Society, Newcastle University, Newcastle upon Tyne, UK; 3 School of Social and Community Medicine, University of Bristol, Bristol, UK; 4 Department of Health Sciences, University of York, York, UK; 5 Department of Psychiatry, University of Melbourne, Melbourne, Victoria, Australia; 6 Centre for Health Economics, University of York, York, UK; 7 Bristol NHS Clinical Commissioning Group, Bristol, UK

**Keywords:** primary care, evaluation, mixed-methods, urgent care

## Abstract

**Introduction:**

Pressure continues to grow on emergency departments in the UK and throughout the world, with declining performance and adverse effects on patient outcome, safety and experience. One proposed solution is to locate general practitioners to work in or alongside the emergency department (GPED). Several GPED models have been introduced, however, evidence of effectiveness is weak. This study aims to evaluate the impact of GPED on patient care, the primary care and acute hospital team and the wider urgent care system.

**Methods and analysis:**

The study will be divided into three work packages (WPs). WP-A; Mapping and Taxonomy: mapping, description and classification of current models of GPED in all emergency departments in England and interviews with key informants to examine the hypotheses that underpin GPED. WP-B; Quantitative Analysis of National Data: measurement of the effectiveness, costs and consequences of the GPED models identified in WP-A, compared with a no-GPED model, using retrospective analysis of Hospital Episode Statistics Data. WP-C; Case Studies: detailed case studies of different GPED models using a mixture of qualitative and quantitative methods including: non-participant observation of clinical care, semistructured interviews with staff, patients and carers; workforce surveys with emergency department staff and analysis of available local routinely collected hospital data. Prospective case study sites will be identified by completing telephone interviews with sites awarded capital funding by the UK government to implement GPED initiatives. The study has a strong patient and public involvement group that has contributed to study design and materials, and which will be closely involved in data interpretation and dissemination.

**Ethics and dissemination:**

The study has been approved by the National Health Service East Midlands—Leicester South Research Ethics Committee: 17/EM/0312. The results of the study will be disseminated through peer-reviewed journals, conferences and a planned programme of knowledge mobilisation.

**Trial registration number:**

ISRCTN51780222.

**Strengths and limitations of this study**

- We will disseminate a comprehensive assessment of general practitioners and emergency departments (GPED) from multiple perspectives to identify models of care which are likely to be most efficient, to maximise clinical and cost-effectiveness, to reduce staff pressure and to improve patient outcome and safety.
- Retrospective analysis of nationally available data and the associated economic analysis will be constrained by the quantity and quality of the available information.
- The mixed-methods approach will allow us to elucidate the aims of GPED, the underlying assumptions about how these aims will be achieved and to assess impacts while considering local context.
- The mixed-methods analysis will also enable the development of recommendations to improve future implementation by identifying challenges and potential solutions.
- GPED models are likely to show significant variation both within and between hospital sites; this may present a challenge to generalisability.

## Introduction

Despite many initiatives to reduce demand, pressure from rising attendance rates continues to grow on emergency departments in the UK, with an associated decrease in performance.^1^ This leads to emergency department crowding, associated with adverse outcomes and increased mortality.^2 3^ There is a clear need to find solutions that reduce the burden on emergency departments and improve patient experience and safety. In England, the ‘Keogh Review’ of urgent and emergency care aims to reduce pressure on emergency departments by treating more patients close to home in primary and community settings.^4^ It includes a recommendation that colocated primary care models should be considered in every emergency department,^5^ however, the optimal model to achieve this has not yet been identified, and evidence for the effectiveness of general practitioners in the emergency department (GPED) is weak both in the UK and internationally.^6–9^ A recent review of primary care services located with emergency departments concluded that there is very little evidence to support this model of care, and that ‘a robust evaluation… is needed to inform future policy’.^10^

Nevertheless, there is an increasing trend to include general practitioners at the hospital front door. A joint report from four Medical Royal Colleges recommended that every emergency department should have a colocated primary care facility.^11^ Estimates of the proportion of emergency department patients that could be managed by a general practitioner vary widely between 15% and 40%.^4 12^ There are a range of models of integration; most involve general practitioner services alongside emergency department staff, with some operating a separate colocated service as a primary care ‘filter’ in front of the emergency department, while others are more integrated with the emergency department team. A three-part taxonomy proposed by the Primary Care Foundation^6^ describes three main operational models: a general practitioner service located alongside or next to the emergency department; general practitioners working at the front of the department screening attendees and either treating or diverting to other places—effectively acting as a filter; general practitioner services fully integrated into a joint operation covering the whole range of primary care and emergency services. Recent evidence suggests that some form of colocation exists in 43% of emergency departments,^13^ however, this is likely to have increased rapidly as a result of policy initiatives to increase colocation, including a capital investment of £100 million made by the UK government in March 2017.^14^

There is also a lack of clarity about the hypothesised mechanism through which locating GPED will reduce pressure on these departments. While it has been suggested that implementing a GPED may have some benefits for patients, the consequences for the healthcare workforce, both general practitioners and hospital staff, have not been studied, particularly when there is real uncertainty as to whether GPED reduces emergency department attendances^15^ and/or emergency admissions.^16^ Some of the apparent impact of GPED on emergency departments may simply be relabelling of the same work, with no real benefits for patients or the National Health Service (NHS). Colocated general practitioner services may further increase demand at hospital sites, transferring the problem of overcrowding from one location to another. In particular, it is not clear what the impact is for general practitioners, who are already overstretched and in short supply, and GPED may not be the best use of their time and skills.^17^ Finally, the cost of implementing and running GPED is a legitimate concern, since employing general practitioners is likely to be more expensive than employing other types of staff.^18^ Although GPED may be effective in reducing emergency admissions, they may not be cost-effective.^9^

For patients as service users, expectations and regard for their care and experience are important considerations. Research indicates that patients may attend the emergency department with non-urgent health problems because of the ease of access or a perceived need for diagnostic tests.^19^ Attendances at emergency departments are a small proportion of all urgent care consultations when compared with general practitioner surgeries in the surrounding area, so small shifts in patient behaviour away from general practice could have major implications for the demand on emergency departments.^20^

Effective evaluation of the different models of GPED, including various approaches to patient triage and streaming, and the extent of integration with existing emergency department services, is essential to inform service development and meet the urgent heath needs of the population. As a result, this issue will remain highly relevant and important to the future needs of healthcare systems. There is a lack of clarity about the intended benefits and mechanisms of GPED, and little evidence to underpin existing hypotheses.

## Research question

What is the impact of GPED on patient care, the primary care and acute hospital team and the wider urgent care system? What is the differential impact of alternative models of GPED?

**Figure 1:**
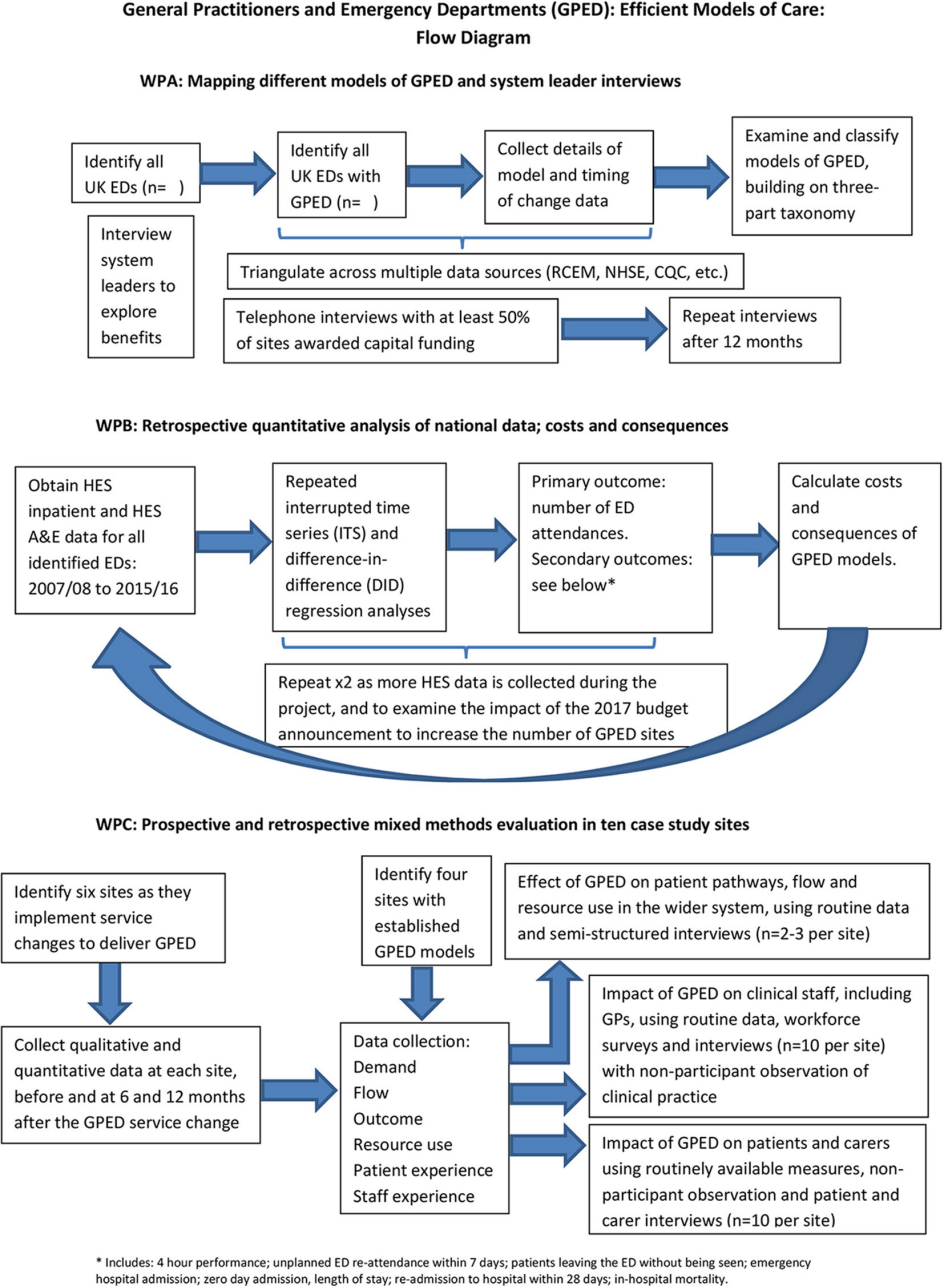
Study flow diagram. A&E, accident and emergency; HES, Hospital Episode Statistics; WP, work package.

The study will use a mixed-methods approach, divided into three work packages (WPs) (figure 1).

This study is an investigation of complex processes and systems and requires the use of both quantitative and qualitative data. Quantitative methods will be used in WP-B to address research questions about causality, effectiveness, costs and consequences. Qualitative methods will be used in WP-A and WP-C to develop in-depth insight into the main models of GPED care; this will include understanding the aims of introducing GPED and the expected outcomes. These hypotheses will vary according to the context of the service being introduced.

This approach draws on the strengths of both quantitative and qualitative paradigms. Qualitative data will enable us to describe the current range of models and the mechanisms by which they operate, and to understand the intended aims of the policy. Quantitative data will allow us to test whether the aims have been achieved, while the synergies arising from a mixed-methods approach to data analysis and interpretation will enable us to explore in depth how and why GPED did or did not work in the way intended in different contexts. This will enable us to make meaningful comparisons across sites and for different models of GPED.

## Methods and analysis

See table 1 for a summary of research objectives, WPs and methods.

**Table 1:** Research objectives, work packages (WPs) and methods

Research objectives	WPs	Methods
1. To map and describe current models of (general practitioners and emergency departments) GPED in England.	WP-A	System leader interviews and documentary analysis.
2. To determine the impact of GPED on patient processes and outcomes including overall attendances, attendances in different components of the local urgent care system, waiting times, emergency admissions, reattendances and mortality.	WP-B WP-C	Retrospective analysis of Hospital Episode Statistics data. Analysis of local data and non-participant observation.
3. To assess the impact of GPED on the casemix of admitted patients by exploring admission rates, including the number and proportion of short stay and zero-day admissions, subject to an examination of coding behaviour by hospital trusts, and any changes that may undermine the reliability of this measure.	WP-B	Retrospective analysis of Hospital Episode Statistics data.
4. To explore the impact of GPED on general practitioners, including turnover, absence, satisfaction, well-being and attitudes to and scope of practice.	WP-C	Mixed-methods approach including workforce surveys and interviews.
5. To explore the impact of GPED on the working patterns and roles of other healthcare professionals in the emergency department, including training, workload, skill mix and expertise.	WP-C	Mixed-methods approach including workforce surveys and interviews.
6. To explore the impact of GPED on local urgent care services, on the wider system including primary care (eg, demand for in-hours and out-of-hours general practitioner appointments), and on the interface between services including patient flow.	WP-C	Mixed-methods approach using secondary data analysis and qualitative techniques.
7. To assess the impact of GPED on patients and carers.	WP-C	Interviews and non-participant observation.
8. To compare resource utilisation and costs of care at emergency department sites with and without GPED, and to compare the costs of different service models.	WP-B	Economic analysis.
9. To prospectively evaluate the current promotion of GPED models of care through collaboration with sites that have bid for capital funding to implement GPED, conducting interviews with identified system leaders and measuring changes in the above parameters over time and as implementation occurs.	WP-C	Prospective mixed-methods case study approach.

### WP-A: mapping and taxonomy

We will map, describe and classify current models of GPED in all emergency departments in England and examine the hypotheses that underpin GPED and its anticipated benefits.

We will work with the Royal College of Emergency Medicine and NHS England to identify, describe and classify current models of general practitioner working in all emergency departments in England, exploring the nature of these colocations, current service configuration, local funding arrangements and the date of commencement of any service change(s). We will triangulate these sources with Care Quality Commission data, direct enquiry to individual sites and relevant data available from other researchers with an interest in this subject area to understand and classify current models of care, building on the three-part taxonomy proposed by the Primary Care Foundation.^6^

We will rank the identified models of care in order of frequency, and anticipate that two or three distinct model types are likely to emerge which we can then describe and examine in more detail through WP-B and WP-C. Work will also be carried out to establish the hypotheses that underpin GPED. We will approach senior clinicians and managers in selected national organisations including NHS England and the Department of Health, inviting them to participate in a semistructured interview that will explore their views on GPED and the potential advantages and disadvantages of this service configuration. We will conduct approximately 10–12 interviews that will be recorded digitally, transcribed verbatim and analysed thematically to identify anticipated benefits and impacts of the main GPED models.

### WP-B: quantitative analysis of national data

We will measure the impact of the GPED models identified in WP-A, compared with a no-GPED model, using retrospective analysis of Hospital Episode Statistics data. We will adopt a quasi-experimental approach using a repeated interrupted time series design and estimate difference-in-difference regression models with closely matched non-GPED sites as controls. A cost-effectiveness analysis will also be conducted on different GPED models based on their estimated effects alongside resource use.

In this WP, we will conduct a quantitative analysis of administrative data to measure the effectiveness, costs and consequences of the most prevalent models of GPED being implemented in hospitals in England.

#### Methods

We will analyse emergency department attendance data, Hospital Episode Statistics inpatient data and Hospital Episode Statistics Accident and Emergency data for the period 2010–2011 to 2015–2016. This analysis will be extended to later periods as more data become available during the lifetime of the project. These data will be gathered to address the primary and secondary outcomes of WP-B, which are listed in box 1.

Box 1 **Summary of primary and secondary outcomes for WP-B**

Primary outcome

- Number of emergency department attendances.

Secondary outcomes

- 4-hour performance.
- Unplanned emergency department reattendance within 7 days.
- Patients leaving the emergency department without being seen.
- Mortality within 28 days of attendance.
- Emergency hospital admission.
- Zero-day admission (subject to an examination of coding behaviour by hospital trusts).

WP, work package.

National data will be used unless there are specific reasons to exclude individual hospitals, for instance, on the grounds of data quality. There is no sampling for this WP, therefore, a sample size calculation is not appropriate.

This WP will also include costs and consequences calculations of different GPED models based on their estimated effects on the outcomes listed in box 1 alongside estimated resource use (eg, general practitioner salaries, incremental change in other staffing levels and costs), all derived from routine administrative datasets and local datasets (WP-C) supplemented by information from WP-A. We will use Personal Social Service Research Unit cost estimates supplemented by local cost estimates to value changes in activity and resource inputs. We will use information on the most common funding arrangements for GPED (from WP-A) to differentiate between costs that fall on hospital and primary care budgets, with the objective of identifying genuine changes in resource utilisation rather than cost shifting.

#### Analysis

We will adopt a quasi-experimental approach, using a combination of interrupted time series and difference-in-difference regression models to identify the causal effect of each GPED model on the primary and secondary WP-B outcomes. We will initially pool all hospitals and GPED types to identify levels and trends before and after implementation, using a multilevel approach. Trends in each hospital and subgroup (based on date of service introduction) will be examined. We will also conduct stratified analysis by GPED model and by early/late adopter status to determine whether there are differences in effects, and as such which model of GPED is most effective.

### WP-C: case studies

Detailed mixed-methods hospital case studies will be completed to examine the effect of GPED on staff, patients, flow and resource use within the wider healthcare system. Prospective case study sites will be purposively sampled from telephone interviews with sites awarded capital funding by the UK government to implement GPED initiatives. Data collection in the case study sites will include:

- Non-participant observation of clinical practice.
- Patient and carer interviews.
- Emergency department data, combined with local data sources relating to the wider urgent care system, including primary care data, where available.
- Longitudinal interview study and staff surveys administered before and after implementation.

In recognition of the limitations of routine data analysis, detailed mixed-methods hospital case studies will be conducted in at least 10 sites that are about to implement (at least 6 sites), or have implemented (at least 4 sites) a GPED model of care, focussing on the main models identified in WP-A. The prospective sites will be evaluated over time; both before, and 6 and 12 months after, the service change.

#### Sampling

The case study sites will be selected purposively to ensure a range of characteristics including type of GPED model (2 or 3 options depending on the findings from WP-A), region of England, hospital characteristics (trauma centre, district hospital, volume), population characteristics (affluent, deprived, urban, rural).

Initially, prospective (newly changing) sites will be identified from the bids that have been submitted to the capital fund established in Spring 2017 to support the rapid introduction of new GPED models of care in emergency departments in England. Working with NHS England, we will identify a system leader in each of the successfully funded sites and invite them to participate in a telephone interview that will identify the local context, planned model, expected benefits and wider impacts. From this information, sites will be selected based on the criteria described above (six sites). In addition, all those who are interviewed from the successfully funded capital bid sites will be contacted again after 12 months to review progress against the originally stated objectives and assess how successful the implementation of GPED has been.

Established sites that have successfully implemented GPED will be identified during WP-A and WP-B, and on the basis of information provided through professional networks and publications. We anticipate recruiting at least four established sites in WP-C.

#### Methods

In all 10 case study sites, a mixture of quantitative and qualitative data will be collected using the following methods.

#### Quantitative data

Quantitative data collection in WP-C will include local routine data and the administration of a staff survey.

##### Local routine data

We will obtain routine data about the number and characteristics of patients who have consulted primary and other urgent care services outside the emergency department, and any available information about other observable characteristics of the local health economy such as patient outcomes and satisfaction, adverse incidents and reports, to provide contextual data that will help us to interpret findings from the case study sites.

##### Staff survey

We will survey staff working in the case study emergency department sites to collate their perceptions of GPED using the NoMAD questionnaire (an implementation measure based on normalisation process theory^21^) alongside standardised and validated measures of work-related experiences and attitudes. The selection of constructs for measurement in this workforce survey will be informed by theoretical models of occupational strain, including the job demands–resources model^22^ and the job demands–control Model.^23^ Specific scales, including measures of job satisfaction and turnover intentions, will be obtained from prior organisational research,^24^ and with reference to major data sources (such as the National General Practitioner Worklife Survey) that will enable comparison.^25^

The survey will be administered to all relevant staff in each case site. In the prospective case sites, the survey will be administered at two time points, to see if perceptions of a new service change over time. Descriptive data from the questionnaire will be linked to data collection and analysis of the qualitative data. In keeping with current recommendations, a basic descriptive analysis of the questionnaire data will be produced to provide an overview of the perceptions of staff within each case study site.

#### Qualitative data

Qualitative data will be collected to ascertain the views and experiences of GPED from the staff and patients at each case study site. This qualitative data collection will comprise non-participant observation and semistructured interviews with healthcare professionals, patients and carers at each of the case study sites.

##### Non-participant observation

Non-participant observation will provide a nuanced insight into how the GPED service model is working in practice within the study case sites. The observations will consist of 2-hour blocks covering different parts of the day/evening and different activities, for example, clinical and non-clinical work, triage, informal interactions and clinical consultations. It is considered that a maximum of 12–16 hours of observations over a 2-week period within each case site will provide sufficient information. Field notes will document everyday working practices, focusing specifically on the nature of the GPED service, how this is operationalised and the response from patients and clinicians. These data will give greater insight into workplace dynamics, relationships, decision-making and the distribution of tasks and responsibilities.

##### Semistructured interviews

Approximately, 10–15 staff will be purposively selected (to include general practitioners, emergency department doctors and nurses of different grades) at each of the sites to participate in an interview regarding their experiences of working within their service model. Interviews will follow a topic guide and will also discuss day-to-day practices, involvement in management/oversight of the GPED service and the participant’s perceived value of GPED.

These interviews will be conducted longitudinally at prospective case study sites, prior to GPED introduction and 12 months after introduction of the new service model.

We will also collect data on the patient/carer experience of GPED, purposively sampling 10–15 patients (and carers where appropriate) who have used the GPED service to participate in a semistructured interview. Patients will be selected to obtain maximum spread based on age, gender and reason for consultation. These interviews will be conducted as soon after attendance as possible, to maximise recall, and will follow a topic guide (box 2).

Box 2 **Key points of topic guides for staff, patient and key informant interviews**

Prospective case study sites: staff interviews

Preimplementation of general practitioners and emergency departments (GPED)

- Expectations of the new model of care.
- Readiness to employ GPED.
- Information on the appropriateness of the preparations made for the introduction of the new service.

Prospective and established case study sites: staff interviews

Postimplementation of GPED

Experience of the new service and the impact on:

- Training/education needs.
- Workload; professional boundaries.
- Job satisfaction/stress.
- Clinical practice and risk management.
- Barriers and facilitators to service introduction.
- Additional topics arising from the local quantitative analysis.

Prospective and established case study sites: key informants

- Advantages and disadvantages of the new model of care.
- Perceived effectiveness of the service model in terms of care provision.
- Impact on staff.
- Barriers and facilitators to successful roll-out.

Patient interviews

- Reasons for attending the emergency department or GPED.
- The influence of GPED on their decision to attend.
- Confidence in GPED compared with an emergency department clinician.
- Impact of GPED on future emergency department and/or general practitioner attendance.

We will also ascertain their experiences of GPED in terms of:

- Quality.
- Advice.
- Referrals and postdischarge care.
- Satisfaction with the service.
- Patients will also be asked to explore which aspects of the service are most important to them, and the barriers/facilitators to service use.

In the prospective sites, we will also conduct semistructured interviews with key informants (commissioners and heads of service) before the GPED service goes live to gain insights into the reasons behind the choice of model, the expectations for the service, how staff have reacted to the plans and the preparatory processes that have taken place to implement the new service. Interviews will be repeated approximately 6 and 12 months following full implementation of GPED.

#### Data analysis

The analysis and interpretation of WP-C will integrate both qualitative and quantitative data and is likely to include the following issues:

- The effect of implementing GPED on patient pathways and flow within the local healthcare system, using non-participant observation and routinely available data.
- The impact of GPED on patients and carers and on healthcare staff using interview data, workforce surveys and routinely available data.
- Barriers and facilitators to the implementation of a GPED model of care, and the development of recommendations to improve future implementation by identifying challenges and potential solutions.

#### Quantitative analysis

The analyses of routine quantitative data at the ‘site’ level will be characterised mainly by descriptive statistics that will complement the qualitative information collected, and which will take due account of any seasonal effects. This approach will enable us to look for potential differences and similarities in views within a case site as well as draw out meaningful comparisons across case sites. Descriptive data from the staff survey will initially inform the purposive selection of participants for qualitative interviews. Further analyses of survey data (eg, regarding job satisfaction and turnover intentions) will be conducted to examine levels and changes across key outcomes over time, and also indicate variables that may explain variance in these outcomes. While regression analyses will be used for the latter purpose, we do not anticipate high levels of statistical power for these analyses, and we will thus focus on reporting of the direction and magnitude of effect size point estimates and 95% CIs.

#### Qualitative analysis

All interviews will be audio recorded digitally and transcribed verbatim. The computer package NVivo will be used to manage the data. Following transcription, the interview material will be organised according to analytical headings using a constant comparison approach. To introduce transparency and a systematic approach, we will engage in: detailed familiarisation; identification and indexing of key themes; contextualising these themes in relation to the broader dataset; interpretation, within the context of theoretical themes relevant to the interview material.^26^

Regular meetings will be held to discuss the emergent themes from the fieldwork material and explore the potential to ‘test’ these in the local quantitative data. The analysis will allow us to gain in-depth insight into the main models of GPED care. This approach will enable us to look for potential differences and similarities in views within a case site as well as draw out meaningful comparisons across case sites and for different models of GPED.

### Patient and public involvement

A patient and public involvement group will be engaged throughout all stages and WPs of the study, to ensure the perspectives of patients and carers are considered fully. We will use a variety of methods to work with the group including face-to-face meetings, email, telephone and video conferencing as appropriate and in keeping with the needs of group members. The patient and public involvement group will be involved in writing the ethics application and developing research instruments and participant information sheets. For WP-A, the group will help to write the interview schedules. Data collection plans for WP-B will also be reviewed. The patient and public involvement group will also provide feedback at various stages of data analysis, particularly during WP-C, and will contribute to dissemination plans.

### Ethics and dissemination

Routine care is not altered by the study, and it therefore does not raise significant ethical issues. All necessary local research governance approvals will be obtained at hospital sites prior to data collection. WP-A includes interviews with NHS staff, and WP-C includes interviews and observations with NHS staff, patients and carers. Appropriate mechanisms to provide written information and informed consent will be instituted for all NHS staff, patient and carer participants. The study will be conducted in accordance with the International Conference for Harmonisation of Good Clinical Practice guidelines (www.ich.org/). The study is registered on the UK Clinical Research Network and the International Standard Randomised Controlled Trial Number Registry.

This study will be disseminated through the Knowledge Mobilisation Team at the Centre for Academic Primary Care, University of Bristol. The study results will be presented at national and international conferences and will be published in international peer-reviewed journals. The study commenced in June 2017 and is expected to run until May 2020, with a total duration of 36 months.

## Discussion

As the number of hospitals implementing GPED increases rapidly, with several competing models in use, the need for definitive evidence regarding the most efficient model of care and best use of scarce resources becomes increasingly urgent. The mixed-methods approach described in this protocol will enable the development of recommendations to improve future implementation by identifying trends, challenges and potential solutions to GPED service change. It is anticipated that the study will generate a comprehensive assessment of GPED from multiple perspectives to identify the models of care that are most likely to be efficient, to maximise clinical and cost-effectiveness, to reduce staff pressure and to improve patient outcome, safety and experience in the UK.

## Supplementary Material

Reviewer comments

Author's manuscript
