# Supplementary material for: General practitioners and emergency departments (GPED)—efficient models of care: a mixed-methods study protocol
Source: BMJ Open. 2018 Oct 3;8(10):e024012. doi: 10.1136/bmjopen-2018-024012 (PMC6194458; doi:10.1136/bmjopen-2018-024012)
Supplement: Author's manuscript [file bmjopen-2018-024012.draft_revisions.pdf]

BMJ Open

BMJ Open is committed to open peer review. As part of this commitment we make the peer review history of every article we publish publicly available.

When an article is published we post the peer reviewers’ comments and the authors’ responses online. We also post the versions of the paper that were used during peer review. These are the versions that the peer review comments apply to.

The versions of the paper that follow are the versions that were submitted during the peer review process. They are not the versions of record or the final published versions. They should not be cited or distributed as the published version of this manuscript.

BMJ Open is an open access journal and the full, final, typeset and author-corrected version of record of the manuscript is available on our site with no access controls, subscription charges or pay-per-view fees (http://bmjopen.bmj.com).

If you have any questions on BMJ Open’s open peer review process please email info.bmjopen@bmj.com

BMJ Open

General Practitioners and Emergency Departments (GPED): Efficient Models of Care. A mixed-methods study protocol.

Journal:	*BMJ Open*
Manuscript ID	bmjopen-2018-024012
Article Type:	Protocol
Date Submitted by the Author:	29-May-2018
Complete List of Authors:	Morton, Katherine; University of the West of England Department of Health and Social Sciences,<br>Voss, Sarah; University of the West of England, Health and Life Sciences<br>Adamson, Joy; Newcastle University<br>Baxter, Helen; University of Bristol, Bristol Medical School: Population Health Sciences<br>Bloor, Karen; University of York, Department of Health Sciences<br>Brandling, Janet; University of the West of England, Health and Life Sciences<br>Cowlishaw , Sean; University of Bristol<br>Doran, Tim; University of York, Health Sciences<br>Gibson, Andy; University of the West of England, Health and Social Sciences<br>Gutacker, Nils; University of York, Centre for Health Economics<br>Liu, Dan; The University of York, Centre for Health Economics<br>Purdy, Sarah; University of Bristol, Centre for Academic Primary Care<br>Roy, Paul; Bristol, North Somerset and South Gloucestershire CCG<br>Salisbury, Chris; University of Bristol, Academic Unit of Primary Health Care<br>Scantlebury, Arabella; Newcastle University, Institute of Health and Society<br>Vaittinen, Anu; Newcastle University Institute for Health and Society<br>Watson, Rose; Newcastle University, Institute of Health and Society<br>Benger, Jonathan; The University Hospitals NHS Foundation trust, Academic Department of Emergency care; The University of the West of England, Faculty of Health & Life Sciences
Keywords:	ACCIDENT & EMERGENCY MEDICINE, PRIMARY CARE, Evaluation, Mixed-methods, Urgent Care

SCHOLARONE™
Manuscripts

General Practitioners and Emergency Departments (GPED): Efficient Models of Care. A mixed-methods study protocol.

Katherine Morton[1]
Sarah Voss[1]
Joy Adamson[2]
Helen Baxter[3]
Karen Bloor[4]
Janet Brandling[1]
Sean Cowlishaw[6]
Tim Doran[4]
Andrew Gibson[1]
Nils Gutacker[5]
Dan Liu[5]
Sarah Purdy[3]
Paul Roy[7]
Christopher Salisbury[3]
Arabella Scantlebury[2]
Anu Vaittinen[2]
Rose Watson[2]
Jonathan Benger[1]

1. Faculty of Health and Life Sciences, University of the West of England, Bristol, United Kingdom.
2. Institute of Health and Society, Newcastle University, Newcastle, United Kingdom.
3. School of Social and Community Medicine, University of Bristol, United Kingdom.
4. Department of Health Sciences, University of York, York, United Kingdom.
5. Centre for Health Economics, University of York, York, United Kingdom.
6. Department of Psychiatry, University of Melbourne, Australia.
7. Bristol NHS Clinical Commissioning Group, Bristol, United Kingdom.

Correspondence to:

Professor Jonathan Benger, University of the West of England, Glenside Campus, Blackberry Hill, Bristol, BS16 1DD, United Kingdom.

Tel: +44(0)117 3421498

E-mail: Jonathan.Benger@uwe.ac.uk

For peer review only

Abstract

Introduction: Pressure continues to grow on Emergency Departments in the United Kingdom and throughout the world, with declining performance and adverse effects on patient outcome, safety and experience. One proposed solution is to locate General Practitioners to work in or alongside the Emergency Department (GPED). Several GPED models have been introduced, however evidence of effectiveness is weak. This study aims to evaluate the impact of GPED on patient care, the primary care and acute hospital team and the wider urgent care system.

Methods and Analysis: The study will be divided into three work-packages (WPs). *WP-A; Mapping and Taxonomy*: mapping, description and classification of current models of GPED in all Emergency Departments in England and interviews with key informants to examine the hypotheses that underpin GPED. *WP-B; Quantitative Analysis of National Data:* measurement of the effectiveness, costs and consequences of the GPED models identified in WP-A, compared to a no-GPED model, using retrospective analysis of Hospital Episode Statistics (HES) Data. *WP-C; Case Studies*: detailed case studies of different GPED models using a mixture of qualitative and quantitative methods including: non-participant observation of clinical care, semi-structured interviews with staff, patients and carers; workforce surveys with Emergency Department staff and analysis of available local routinely collected hospital data. Prospective case study sites will be identified by completing telephone interviews with sites awarded capital funding by the United Kingdom government to implement GPED initiatives. The study has a strong patient and public involvement group that has contributed to study design and materials, and which will be closely involved in data interpretation and dissemination.

Ethics and dissemination: The study has been approved by the National Health Service East Midlands- Leicester South Research Ethics Committee: 17/EM/0312. The results of the study will be disseminated through peer-reviewed journals, conferences and a planned programme of knowledge mobilisation.

Trial Registration: ISRCTN: 51780222.

For peer review only

Strengths and Limitations of this Study

- We will disseminate a comprehensive assessment of GPED from multiple perspectives to identify models of care which are likely to be most efficient, to maximise clinical and cost-effectiveness, to reduce staff pressure and to improve patient outcome and safety.
- Retrospective analysis of nationally available data, and the associated economic analysis, will be constrained by the quantity and quality of the available information.
- The mixed-methods approach will allow us to elucidate the aims of GPED, the underlying assumptions about how these aims will be achieved, and to assess impacts while considering local context.
- The mixed-methods analysis will also enable the development of recommendations to improve future implementation by identifying challenges and potential solutions.
- GPED models are likely to show significant variation both within and between hospital sites; this may present a challenge to generalisability.

Introduction

Despite many initiatives to reduce demand, pressure from rising attendance rates continues to grow on Emergency Departments in the United Kingdom, with an associated decrease in performance.[1] This leads to Emergency Department crowding, associated with adverse outcomes and increased mortality.[2,3] There is a clear need to find solutions that reduce the burden on Emergency Departments and improve patient experience and safety. In England, the "Keogh Review" of urgent and emergency care aims to reduce pressure on Emergency Departments by treating more patients close to home in primary and community settings.[4] It includes a recommendation that co-located primary care models should be considered in every Emergency Department,[5] however, the optimal model to achieve this has not yet been identified, and evidence for the effectiveness of General Practitioners in the Emergency Department is weak both in the United Kingdom and internationally.[6-9] A recent review of primary care services located with Emergency Departments concluded that there is very little evidence to support this model of care, and that "a robust evaluation… is needed to inform future policy".[10]

Nevertheless, there is an increasing trend to include General Practitioners at the hospital front door. A joint report from four Medical Royal Colleges recommended that every Emergency Department should have a co-located primary care facility.[11] Estimates of the proportion of Emergency Department patients that could be managed by a General Practitioner vary widely between 15% and 40%.[12,13] There are a range of models of integration; most involve General Practitioner services alongside Emergency Department staff, with some operating a separate co-located service as a primary care "filter" in front of the Emergency Department, while others are more integrated with the Emergency Department team. A three-part taxonomy proposed by the Primary Care Foundation [6]

For peer review only

describes three main operational models: a General Practitioner service located alongside or next to the emergency department; General Practitioners working at the front of the department screening attendees and either treating or diverting to other places – effectively acting as a filter; General Practitioner services fully integrated into a joint operation covering the whole range of primary care and emergency services. Recent evidence suggests that some form of co-location exists in 43% of Emergency Departments,[14] however this is likely to have increased rapidly as a result of policy initiatives to increase co-location, including a capital investment of £100 million made by the United Kingdom government in March 2017.[15]

There is also a lack of clarity about the hypothesised mechanism through which locating General Practitioners in Emergency Department will reduce pressure on these departments. Whilst it is has been suggested that implementing a General Practitioner in or alongside the Emergency Department (GPED) may have some benefits for patients, the consequences for the healthcare workforce, both General Practitioners and hospital staff, have not been studied, particularly when there is real uncertainty as to whether GPED reduces Emergency Department attendances,[16] and/or emergency admissions.[17] Some of the apparent impact of GPED on Emergency Departments may simply be re-labelling of the same work, with no real benefits for patients or the National Health Service. Co-located General Practitioner services may further increase demand at hospital sites, transferring the problem of over-crowding from one location to another. In particular, it is not clear what the impact is for General Practitioners, who are already overstretched and in short supply, and GPED may not be the best use of their time and skills.[18] Finally, the cost of implementing and running GPED is a legitimate concern, since employing General Practitioners is likely to be more expensive than employing other types of staff.[19] Although General Practitioners in the Emergency Department may be effective in reducing emergency admissions, they may not be cost effective.[9]

For patients as service users, expectations and regard for their care and experience are important considerations. Research indicates that patients may attend the Emergency Department with non-urgent health problems because of the ease of access or a perceived need for diagnostic tests.[20] Attendances at Emergency Departments are a small proportion of all urgent care consultations when compared to General Practitioner surgeries in the surrounding area, so small shifts in patient behaviour away from General Practice could have major implications for the demand on Emergency Departments.[21]

Effective evaluation of the different models of GPED, including various approaches to patient triage and streaming, and the extent of integration with existing Emergency Department services, is essential to inform service development and meet the urgent heath needs of the population. As a result, this issue will remain highly relevant and important to the future needs of healthcare systems. There is a lack of clarity about the intended benefits and mechanisms of GPED, and little evidence to underpin existing hypotheses.

For peer review only

Research question

What is the impact of General Practitioners working in or alongside the Emergency Department (GPED) on patient care, the primary care and acute hospital team and the wider urgent care system? What is the differential impact of alternative models of GPED?

The study will use a mixed-methods approach, divided into three Work Packages (WPs) (Figure 1).

This study is an investigation of complex processes and systems and requires the use of both quantitative and qualitative data. Quantitative methods will be used in WP-B to address research questions about causality, effectiveness, costs and consequences. Qualitative methods will be used in WP-A and WP-C to develop in-depth insight into the main models of GPED care; this will include understanding the aims of introducing GPED and the expected outcomes. These hypotheses will vary according to the context of the service being introduced.

This approach draws upon the strengths of both quantitative and qualitative paradigms. Qualitative data will enable us to describe the current range of models and the mechanisms by which they operate, and to understand the intended aims of the policy. Quantitative data will allow us to test whether the aims have been achieved, whilst the synergies arising from a mixed methods approach to data analysis and interpretation will enable us to explore in depth how and why GPED did or did not work in the way intended in different contexts. This will enable us to make meaningful comparisons across sites, and for different models of GPED.

Figure One: Study Flow Diagram

For peer review only

Methods and Analysis

Table One: Summary of Research Objectives, Work Packages and Methods

**Research Objectives**	**Work Packages**	**Methods**
1. To map and describe current models of GPED in England	**WP-A**	System leader interviews and documentary analysis
2. To determine the impact of GPED on patient processes and outcomes including overall attendances, attendances in different components of the local urgent care system, waiting times, emergency admissions, re-attendances and mortality	**WP-B**<br><br>**WP-C**	Retrospective analysis of Hospital Episode Statistics data<br>Analysis of local data and<br>non-participant observation
3. To assess the impact of GPED on the case-mix of admitted patients by exploring admission rates, including the number and proportion of short stay and zero day admissions, subject to an examination of coding behaviour by hospital trusts, and any changes that may undermine the reliability of this measure	**WP-B**	Retrospective analysis of Hospital Episode Statistics data
4. To explore the impact of GPED on General Practitioners, including turnover, absence, satisfaction, well-being and attitudes to and scope of practice	**WP-C**	Mixed-methods approach including workforce surveys and interviews
5. To explore the impact of GPED on the working patterns and roles of other health care professionals in the Emergency Department, including training, workload, skill-mix and expertise	**WP-C**	Mixed-methods approach including workforce surveys and interviews
6. To explore the impact of GPED on local urgent care services, on the wider system including primary care (e.g. demand for in-hours and out-of-hours General Practitioner appointments), and on the interface between services including patient flow	**WP-C**	Mixed methods approach using secondary data analysis and qualitative techniques
7. To assess the impact of GPED on patients and carers	**WP-C**	Interviews and non-participant observation
8. To compare resource utilisation and costs of care at Emergency Department sites with and without General Practitioners in or alongside the Emergency Department, and to compare the costs of different service models.	**WP-B**	Economic analysis
9. To prospectively evaluate the current promotion of GPED models of care through collaboration with sites that have bid for capital funding to implement GPED, conducting interviews with identified system leaders and measuring changes in the above parameters over time and as implementation occurs	**WP-C**	Prospective mixed-methods case study approach

For peer review only - http://bmjopen.bmj.com/site/about/guidelines.xhtml

WP-A: Mapping and Taxonomy

We will map, describe and classify current models of GPED in all Emergency Departments in England and examine the hypotheses that underpin GPED and its anticipated benefits.

We will work with the Royal College of Emergency Medicine and NHS England to identify, describe and classify current models of General Practitioner working in all Emergency Departments in England, exploring the nature of these co-locations, current service configuration, local funding arrangements and the date of commencement of any service change(s). We will triangulate these sources with Care Quality Commission data, direct enquiry to individual sites and relevant data available from other researchers with an interest in this subject area to understand and classify current models of care, building on the three-part taxonomy proposed by the Primary Care Foundation.[6]

We will rank the identified models of care in order of frequency, and anticipate that two or three distinct model types are likely to emerge which we can then describe and examine in more detail through WP-B and WP-C. Work will also be carried out to establish the hypotheses that underpin GPED. We will approach senior clinicians and managers in selected national organisations including NHS England and the Department of Health, inviting them to participate in a semi-structured interview that will explore their views on GPED and the potential advantages and disadvantages of this service configuration. We will conduct approximately 10-12 interviews that will be recorded digitally, transcribed verbatim and analysed thematically to identify anticipated benefits and impacts of the main GPED models.

WP-B: Quantitative Analysis of National Data

We will measure the impact of the GPED models identified in WP-A, compared to a no-GPED model, using retrospective analysis of Hospital Episode Statistics data. We will adopt a quasi-experimental approach using a repeated interrupted time series design and estimate difference-in-difference regression models with closely matched non-GPED sites as controls. A cost-effectiveness analysis will also be conducted on different GPED models based on their estimated effects alongside resource use.

In this work package we will conduct a quantitative analysis of administrative data to measure the effectiveness, costs and consequences of the most prevalent models of GPED being implemented in hospitals in England.

Methods

We will analyse Emergency Department attendance data, Hospital Episode Statistics inpatient data, and Hospital Episode Statistics Accident and Emergency data for the period

For peer review only

2010-11 to 2015-16. This analysis will be extended to later periods as more data become available during the lifetime of the project. These data will be gathered to address the primary and secondary outcomes of WP-B, which are listed in Table Two.

Table Two: Summary of Primary and Secondary outcomes for Work Package B

**Primary Outcome**
Number of Emergency Department attendances
**Secondary Outcomes**
4 hour performance
Unplanned Emergency Department re-attendance within 7 days
Patients leaving the Emergency Department without being seen
Mortality within 28 days of attendance
Emergency hospital admission
Zero day admission (subject to an examination of coding behaviour by hospital Trusts)

National data will be used unless there are specific reasons to exclude individual hospitals, for instance on the grounds of data quality. There is no sampling for this work package, therefore a sample size calculation is not appropriate.

This work package will also include costs and consequences calculations of different GPED models based on their estimated effects on the outcomes listed in Table 2 alongside estimated resource use (e.g. General Practitioner salaries, incremental change in other staffing levels and costs), all derived from routine administrative datasets and local datasets (WP-C) supplemented by information from WP-A. We will use Personal Social Service Research Unit cost estimates supplemented by local cost estimates to value changes in activity and resource inputs. We will use information on the most common funding arrangements for GPED (from WP-A) to differentiate between costs that fall on hospital and primary care budgets, with the objective of identifying genuine changes in resource utilisation rather than cost shifting.

Analysis

We will adopt a quasi-experimental approach, using a combination of interrupted time series and difference-in-difference regression models to identify the causal effect of each GPED model on the primary and secondary WP-B outcomes. We will initially pool all hospitals and GPED types to identify levels and trends before and after implementation, using a multi-level approach. Trends in each hospital and subgroup (based on date of service introduction) will be examined. We will also conduct stratified analysis by GPED model and by early/late adopter status to determine whether there are differences in effects, and as such which model of GPED is most effective.

For peer review only

WP-C: Case Studies

Detailed mixed-methods hospital case studies will be completed to examine the effect of GPED on staff, patients, flow and resource use within the wider healthcare system. Prospective case study sites will be purposively sampled from telephone interviews with sites awarded capital funding by the United Kingdom government to implement GPED initiatives. Data collection in the case study sites will include:

- Non-participant observation of clinical practice
- Patient and carer interviews
- Emergency Department data, combined with local data sources relating to the wider urgent care system, including primary care data, where available
- Longitudinal interview study and staff surveys administered before and after implementation

In recognition of the limitations of routine data analysis, detailed mixed-methods hospital case studies will be conducted in at least ten sites that are about to implement (at least six sites), or have implemented (at least four sites) a GPED model of care, focussing on the main models identified in WP-A. The prospective sites will be evaluated over time; both before, and 6 and 12 months after, the service change.

Sampling

The case study sites will be selected purposively to ensure a range of characteristics including type of GPED model (2 or 3 options depending on the findings from WP-A), region of England, hospital characteristics (trauma centre, district hospital, volume), population characteristics (affluent, deprived, urban, rural).

Initially, prospective (newly changing) sites will be identified from the bids that have been submitted to the capital fund established in Spring 2017 to support the rapid introduction of new GPED models of care in Emergency Departments in England. Working with NHS England, we will identify a system leader in each of the successfully funded sites and invite them to participate in a telephone interview that will identify the local context, planned model, expected benefits and wider impacts. From this information, sites will be selected based on the criteria described above (six sites). In addition, all those who are interviewed from the successfully funded capital bid sites will be contacted again after 12 months to review progress against the originally stated objectives and assess how successful the implementation of GPED has been.

For peer review only

Established sites that have successfully implemented GPED will be identified during WP-A and WP-B, and on the basis of information provided through professional networks and publications. We anticipate recruiting at least four established sites in WP-C.

Methods

In all ten case study sites a mixture of quantitative and qualitative data will be collected using the following methods.

Quantitative Data

Quantitative data collection in WP-C will include local routine data and the administration of a staff survey.

Local Routine Data

We will obtain routine data about the number and characteristics of patients who have consulted primary and other urgent care services outside the Emergency Department, and any available information about other observable characteristics of the local health economy such as patient outcomes and satisfaction, adverse incidents and reports, to provide contextual data that will help us to interpret findings from the case study sites.

Staff Survey

We will survey staff working in the case study Emergency Department sites to collate their perceptions of GPED using the NoMAD questionnaire (an implementation measure based on Normalization Process Theory (NPT)[22]) alongside standardised and validated measures of work-related experiences and attitudes. The selection of constructs for measurement in this workforce survey will be informed by theoretical models of occupational strain, including the Job Demands-Resources Model,[23] and the Job Demands-Control Model.[24] Specific scales, including measures of job satisfaction and turnover intentions, will be obtained from prior organisational research,[25] and with reference to major data sources (such as the National General Practitioner Worklife Survey) that will enable comparison.[26]

The survey will be administered to all relevant staff in each case site. In the prospective case sites, the survey will be administered at two time points, to see if perceptions of a new service change over time. Descriptive data from the questionnaire will be linked to data collection and analysis of the qualitative data. In keeping with current recommendations, a basic descriptive analysis of the questionnaire data will be produced to provide an overview of the perceptions of staff within each case study site.

Qualitative Data

For peer review only

Qualitative data will be collected to ascertain the views and experiences of GPED from the staff and patients at each case study site. This qualitative data collection will comprise non-participant observation and semi-structured interviews with healthcare professionals, patients and carers at each of the case-study sites.

Non-participant Observation

Non-participant observation will provide a nuanced insight into how the GPED service model is working in practice within the study case sites. The observations will consist of two-hour blocks covering different parts of the day/evening and different activities, e.g. clinical and non-clinical work, triage, informal interactions and clinical consultations. It is considered that a maximum of 12-16 hours of observations over a two-week period within each case site will provide sufficient information. Field notes will document everyday working practices, focusing specifically on the nature of the GPED service, how this is operationalised and the response from patients and clinicians. These data will give greater insight into workplace dynamics, relationships, decision-making and the distribution of tasks and responsibilities.

Semi-structured interviews

Approximately 10-15 staff will be purposively selected (to include General Practitioners, Emergency Department doctors and nurses of different grades) at each of the sites to participate in an interview regarding their experiences of working within their service model. Interviews will follow a topic guide and will also discuss day-to-day practices, involvement in management/oversight of the GPED service and the participant's perceived value of GPED.

These interviews will be conducted longitudinally at prospective case study sites, prior to GPED introduction and 12 months after introduction of the new service model.

We will also collect data on the patient/carer experience of GPED, purposively sampling 10-15 patients (and carers where appropriate) who have used the GPED service to participate in a semi-structured interview. Patients will be selected to obtain maximum spread based on age, gender, and reason for consultation. These interviews will be conducted as soon after attendance as possible, to maximize recall, and will follow a topic guide (Figure Two).

In the prospective sites we will also conduct semi-structured interviews with key informants (commissioners and heads of service) before the GPED service goes live to gain insights into the reasons behind the choice of model, the expectations for the service, how staff have reacted to the plans and the preparatory processes that have taken place to implement the new service. Interviews will be repeated approximately 6 and 12 months following full implementation of GPED.

For peer review only - http://bmjopen.bmj.com/site/about/guidelines.xhtml

Table Three: Key points of topic guides for staff, patient and key informant interviews

Prospective case study sites: staff interviews

Pre-implementation of GPED

- expectations of the new model of care
- readiness to employ GPED
- information on the appropriateness of the preparations made for the introduction of the new service

Prospective and established case study sites: staff interviews

Post-implementation of GPED

Experience of the new service and the impact on:

- training/education needs
- workload; professional boundaries
- job satisfaction/stress
- clinical practice and risk management
- barriers and facilitators to service introduction
- additional topics arising from the local quantitative analysis.

Prospective and established case study sites: key informants

- advantages and disadvantages of the new model of care
- perceived effectiveness of the service model in terms of care provision
- impact on staff
- barriers and facilitators to successful roll out.

Patient interviews

- reasons for attending the Emergency Department or GPED
- the influence of GPED on their decision to attend
- confidence in GPED compared to an Emergency Department clinician
- impact of GPED on future Emergency Department and/or General Practitioner attendance.

We will also ascertain their experiences of GPED in terms of:

- quality
- advice
- referrals and post-discharge care
- satisfaction with the service
- patients will also be asked to explore which aspects of the service are most important to them, and the barriers/facilitators to service use.

For peer review only

Data Analysis

The analysis and interpretation of WP-C will integrate both qualitative and quantitative data and is likely to include the following issues:

- The effect of implementing GPED on patient pathways and flow within the local healthcare system, using non-participant observation and routinely available data;
- The impact of GPED on patients and carers and on healthcare staff using interview data, workforce surveys and routinely available data;

- Barriers and facilitators to the implementation of a GPED model of care, and the development of recommendations to improve future implementation by identifying challenges and potential solutions.

Quantitative Analysis

The analyses of routine quantitative data at the 'site' level will be characterised mainly by descriptive statistics that will complement the qualitative information collected, and which will take due account of any seasonal effects. This approach will enable us to look for potential differences and similarities in views within a case site as well as draw out meaningful comparisons across case sites. Descriptive data from the staff survey will initially inform the purposive selection of participants for qualitative interviews. Further analyses of survey data (e.g., regarding job satisfaction and turnover intentions) will be conducted to examine levels and changes across key outcomes over time, and also indicate variables that may explain variance in these outcomes. While regression analyses will be used for the latter purpose, we do not anticipate high levels of statistical power for these analyses, and we will thus focus on reporting of the direction and magnitude of effect size point estimates and 95% Confidence Intervals.

Qualitative Analysis

All interviews will be audio recorded digitally and transcribed verbatim. The computer package NVIVO will be used to manage the data. Following transcription, the interview material will be organised according to analytical headings using a constant comparison approach. To introduce transparency and a systematic approach we will engage in: detailed familiarisation; identification and indexing of key themes; contextualising these themes in relation to the broader dataset; interpretation, within the context of theoretical themes relevant to the interview material.[27]

Regular meetings will be held to discuss the emergent themes from the fieldwork material and explore the potential to 'test' these in the local quantitative data. The analysis will allow us to gain in-depth insight into the main models of GPED care. This approach will enable us to look for potential differences and similarities in views within a case site as well as draw out meaningful comparisons across case sites and for different models of GPED.

Patient and Public Involvement

A Patient and Public Involvement Group will be engaged throughout all stages and WPs of the study, to ensure the perspectives of patients and carers are considered fully. As far as possible, we involve people who have a variety of experiences of using ED departments. e.g people with conditions who make them more regular users of ED Departments as well as more infrequent users. We will use a variety of methods to work with the group including face to face meetings, e-mail, telephone and video conferencing as appropriate and in

For peer review only

keeping with the needs of group members. The Patient and Public Involvement Group will be involved in writing the ethics application and developing research instruments and participant information sheets. For WP-A, the Group will help to write the interview schedules. Data collection plans for WP-B will also be reviewed. The Patient and Public Involvement group will also provide feedback at various stages of data analysis, particularly during WP-C, where we plan to run workshops with the aim of developing our data analysis jointly with PPI group. The group will also contribute to dissemination plans.

Ethics and dissemination

Routine care is not altered by the study, and it therefore does not raise significant ethical issues. All necessary local research governance approvals will be obtained at hospital sites prior to data collection. WP-A includes interviews with NHS staff, and WP-C includes interviews and observations with NHS staff, patients and carers. Appropriate mechanisms to provide written information and informed consent will be instituted for all NHS staff, patient and carer participants. The study will be conducted in accordance with the International Conference for Harmonisation of Good Clinical Practice guidelines (www.ich.org/). The study is registered on the United Kingdom Clinical Research Network and the International Standard Randomised Controlled Trial Number Registry.

This study will be disseminated through the Knowledge Mobilisation Team at the Centre for Academic Primary Care, University of Bristol. The study results will be presented at national and international conferences and will be published in international peer-reviewed journals. The study commenced in June 2017 and is expected to run until May 2020, with a total duration of 36 months.

Discussion

As the number of hospitals implementing GPED increases rapidly, with several competing models in use, the need for definitive evidence regarding the most efficient model of care, and best use of scarce resources, becomes increasingly urgent. The mixed-methods approach described in this protocol will enable the development of recommendations to improve future implementation by identifying trends, challenges and potential solutions to GPED service change. It is anticipated that the study will generate a comprehensive assessment of GPED from multiple perspectives to identify the models of care that are most likely to be efficient, to maximise clinical and cost-effectiveness, to reduce staff pressure and to improve patient outcome, safety and experience in the United Kingdom.

For peer review only

Authors' contributions: The GPED study team, and the contribution made by each individual, are described in the following statement. The Chief Investigator and study guarantor is Jonathan Benger. The research question was developed and the study was designed by Jonathan Benger, Joy Adamson, Helen Baxter, Karen Bloor, Sean Cowlishaw, Tim Doran, Andrew Gibson, Nils Gutacker, Sarah Purdy, Chris Salisbury and Sarah Voss. The study protocol was developed and refined by Jonathan Benger, Katherine Morton, Sarah Voss, Joy Adamson, Helen Baxter, Karen Bloor, Janet Brandling, Sean Cowlishaw, Tim Doran, Andrew Gibson, Nils Gutacker, Dan Liu, Sarah Purdy, Paul Roy, Chris Salisbury, Arabella Scantlebury, Anu Vaittinen, Rose Watson and Sarah Voss. Financial and contract management is provided by Paul Roy. All authors have reviewed, revised and approved the final manuscript.

Funding statement: This work was supported by the National Institute of Health Research (NIHR) Health services & Delivery Research (HS&DR) Programme, project number 15/145/06.

Disclaimer: The views and opinions expressed therein are those of the authors and do not necessarily reflect those of the Health Services & Delivery Research Programme., NIHR, NHS or the Department of Health.

Competing Interests Statement: Jonathan Benger is seconded part-time to the post of National Clinical Director for Urgent Care at NHS England. All other authors declare no conflict of interest.

Ethics approval: Approval for the study has been obtained from the Health Research Authority (HRA). The protocol was reviewed and received a favourable opinion from the National Health Service (NHS) East Midlands- Leicester South Research Ethics Committee- REC: 17/EM/0312.

Provenance and peer review: Not commissioned; peer reviewed for ethics and funding approval prior to submission.

Open Access Statement: This is an open access article distributed in accordance with the terms of the creative commons attribution (CC BY 4.0) license, which permits others to distribute, remix and adapt and build upon this work for commercial use, provided the original work is properly cited.

For peer review only

References

1. Monitor. A&E delays: why did patients wait longer last winter? Monitor, London: 2015.

2. Collis J. Adverse effects of overcrowding on patient experience and care. *Emerg Nurse* 2010;18(8):34- 39.

3. Richardson DB. Increase in patient mortality at 10 days associated with emergency department overcrowding. *Med J Aust* 2006;184:213-6.

4. NHS England. Transforming urgent and emergency care services in England. Urgent and Emergency Care Review End of Phase 1 Report. NHS England, London: 2013.

5. UEC Review Team and ECIST. Transforming urgent and emergency care services in England. Safer, faster, better: good practice in delivering urgent and emergency care. A guide for local health and social care communities. NHS England, London: 2015.

6. Carson D, Clay H, Stern R. Primary Care and Emergency Departments: Report from the Primary Care Foundation. Primary Care Foundation, London: 2010.

7. Berchet C. OECD Working Paper No. 83. Emergency care services: trends, drivers and interventions to reduce demand. OECD, Paris: 2015.

8. Khangura JK, Flodgren G, Perera R, Rowe BH, Shepperd S. Does employing general practitioners to provide care for patients with non-urgent problems in emergency departments decrease resource use and costs? Cochrane Database of Systematic Reviews 2012, Issue 11. Art. No.: CD002097. DOI: 10.1002/14651858.CD002097.pub3.

9. Purdy S. Avoiding hospital admissions. What does the research evidence say? The King's Fund, London: 2010.

10.Ramlakhan S, Mason S, O'Keeffe C, Ramtahal A, Ablard S. Primary care services located with EDs: a review of effectiveness. *Emerg Med J* 2016. doi:10.1136/emermed-2015-204900.

11.Anonymous. Acute and emergency care: prescribing the remedy. Royal College of Emergency Medicine, London: 2014.

12.Royal College of Emergency Medicine and the Patients Association. Time to act: urgent care and A&E – the patient perspective. RCEM, London: 2015.

13.NHS England. Transforming urgent and emergency care services in England. Urgent and Emergency Care Review End of Phase 1 Report. NHS England, London: 2013.

14.Royal College of Emergency Medicine and Urgent Health UK. The Royal College of Emergency Medicine and Urgent Health UK call for the colocation of urgent care services with A&E departments (press statement, 4th November 2015).

15. Lacobucci, G. All A&Es must have GP led triage. *BMJ* 2017;356:j1270.

For peer review only

16.Salisbury C, Chalder M, Hollinghurst S, et al. The impact of NHS walk-in centres on A&E services. Final report. University of Bristol, Bristol: 2006.

17.O'Dowd, A. GPs in A&E could help tackle unsustainable rise in emergency hospital admissions. *BMJ* 2010;341:c3618.

18.Royal College of General Practitioners. RCGP response to College of Emergency Medicine report. RCGP, London: 2015.

19. Monitor. Walk-in centre review: final report and recommendations. Monitor, London: 2014.

20.Giesen P, Franssen E, Mokkink H, van den Bosch W, van Vugt A, Grol, R. Patients either contacting a general practice cooperative or accident and emergency department out of hours: a comparison. *Emerg Med J* 2006;23(9):731-734.

21.Huibers L, Thijssen W, Koetsenruijter J, Giesen P, Grol R, Wensing M. GP cooperative and emergency department: an exploration of patient flows. *J Eval Clin Pract* 2013;19(2):243-9.

22.Finch TL, Girling M, May CR, Mair FS, Murray E, Treweek S, et al. NoMAD: Implementation measure based on Normalization Process Theory. [Measurement instrument].2015. Available at: http://www.normalizationprocess.org

23.Bakker AB, Demerouti E. The job demands-resources model: State of the art. *Journal of Managerial Psychology* 2007;22:309-328.

24.Karasek Jr, RA. Job demands, job decision latitude, and mental strain: Implications for job redesign. *Administrative Science Quarterly* 1979:285-308.

25.Van Dick R, Christ O, Stellmacher J, Wagner U, Ahlswede O, Grubba C, et al. Should I stay or should I go? Explaining turnover intentions with organizational identification and job satisfaction. *British Journal of Management* 2004;15:351-360.

26.Hann M, McDonald J, Checkland K, Coleman A, Gravelle H, Sibbald B, et al. Seventh national GP worklife survey. University of Manchester; Manchester: 2013.

27. Braun V, Clarke V. Using thematic analysis in psychology. *Qualitative Research in Psychology* 2006;3(2):77-101.

For peer review only

Figure One: Study Flow Diagram

For peer review only

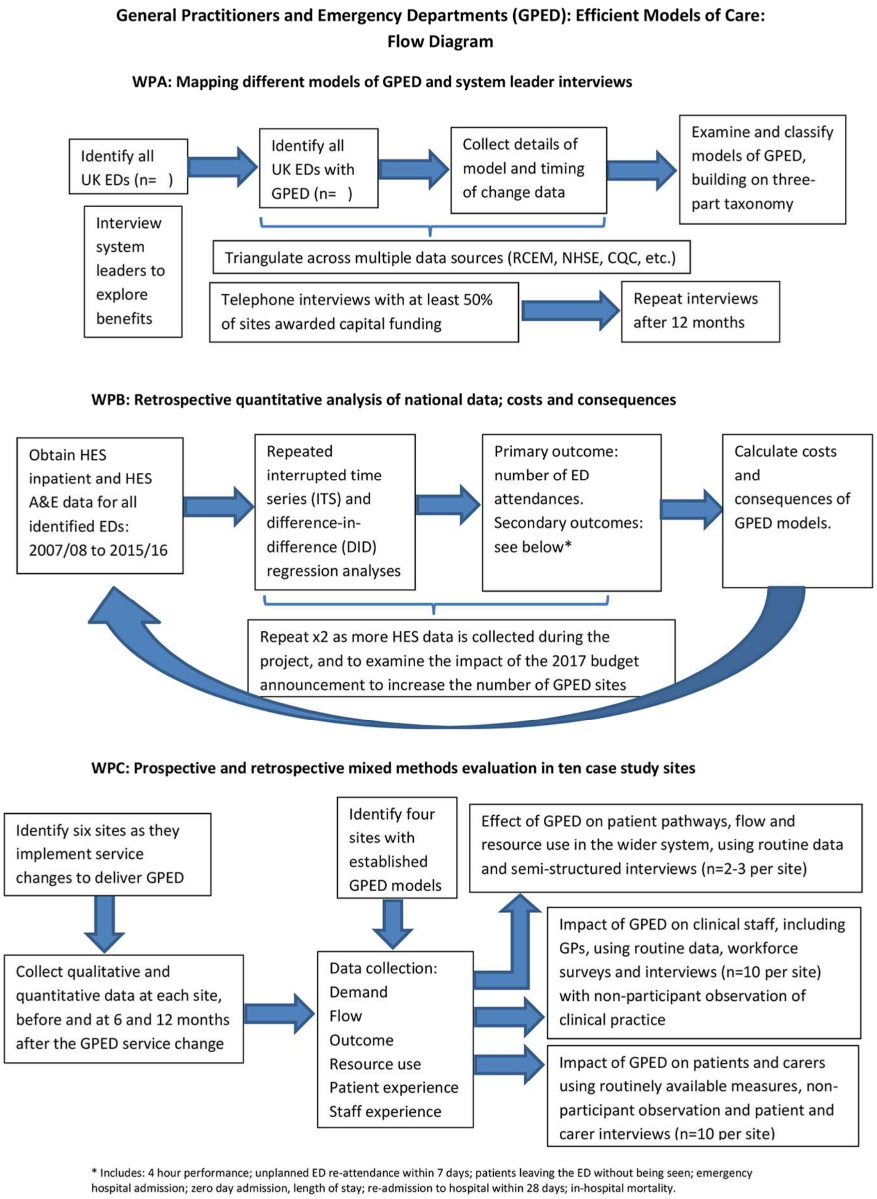

Figure One: Study Flow Diagram

119x165mm (300 x 300 DPI)

BMJ Open

General Practitioners and Emergency Departments (GPED): Efficient Models of Care. A mixed-methods study protocol.

Journal:	*BMJ Open*
Manuscript ID	bmjopen-2018-024012.R1
Article Type:	Protocol
Date Submitted by the Author:	13-Jul-2018
Complete List of Authors:	Morton, Katherine; University of the West of England Department of Health and Social Sciences,<br>Voss, Sarah; University of the West of England, Health and Life Sciences<br>Adamson, Joy; Newcastle University<br>Baxter, Helen; University of Bristol, Bristol Medical School: Population Health Sciences<br>Bloor, Karen; University of York, Department of Health Sciences<br>Brandling, Janet; University of the West of England, Health and Life Sciences<br>Cowlishaw , Sean; University of Bristol<br>Doran, Tim; University of York, Health Sciences<br>Gibson, Andy; University of the West of England, Health and Social Sciences<br>Gutacker, Nils; University of York, Centre for Health Economics<br>Liu, Dan; The University of York, Centre for Health Economics<br>Purdy, Sarah; University of Bristol, Centre for Academic Primary Care<br>Roy, Paul; Bristol, North Somerset and South Gloucestershire CCG<br>Salisbury, Chris; University of Bristol, Academic Unit of Primary Health Care<br>Scantlebury, Arabella; Newcastle University, Institute of Health and Society<br>Vaittinen, Anu; Newcastle University Institute for Health and Society<br>Watson, Rose; Newcastle University, Institute of Health and Society<br>Benger, Jonathan; The University Hospitals NHS Foundation trust, Academic Department of Emergency care; The University of the West of England, Faculty of Health & Life Sciences
**Primary Subject Heading**:	Emergency medicine
Secondary Subject Heading:	Health services research
Keywords:	ACCIDENT & EMERGENCY MEDICINE, PRIMARY CARE, Evaluation, Mixed-methods, Urgent Care

SCHOLARONE™
Manuscripts

General Practitioners and Emergency Departments (GPED): Efficient Models of Care. A mixed-methods study protocol.

Katherine Morton[1]
Sarah Voss[1]
Joy Adamson[2]
Helen Baxter[3]
Karen Bloor[4]
Janet Brandling[1]
Sean Cowlishaw[6]
Tim Doran[4]
Andrew Gibson[1]
Nils Gutacker[5]
Dan Liu[5]
Sarah Purdy[3]
Paul Roy[7]
Christopher Salisbury[3]
Arabella Scantlebury[2]
Anu Vaittinen[2]
Rose Watson[2]
Jonathan Benger[1]

1. Faculty of Health and Life Sciences, University of the West of England, Bristol, United Kingdom.
2. Institute of Health and Society, Newcastle University, Newcastle, United Kingdom.
3. School of Social and Community Medicine, University of Bristol, United Kingdom.
4. Department of Health Sciences, University of York, York, United Kingdom.
5. Centre for Health Economics, University of York, York, United Kingdom.
6. Department of Psychiatry, University of Melbourne, Australia.
7. Bristol NHS Clinical Commissioning Group, Bristol, United Kingdom.

Correspondence to:

Professor Jonathan Benger, University of the West of England, Glenside Campus, Blackberry Hill, Bristol, BS16 1DD, United Kingdom.

Tel: +44(0)117 3421498

Email: Jonathan.Benger@uwe.ac.uk

For peer review only

Abstract

Introduction: Pressure continues to grow on Emergency Departments in the United Kingdom and throughout the world, with declining performance and adverse effects on patient outcome, safety and experience. One proposed solution is to locate General Practitioners to work in or alongside the Emergency Department (GPED). Several GPED models have been introduced, however evidence of effectiveness is weak. This study aims to evaluate the impact of GPED on patient care, the primary care and acute hospital team and the wider urgent care system.

Methods and Analysis: The study will be divided into three work-packages (WPs). *WP-A; Mapping and Taxonomy*: mapping, description and classification of current models of GPED in all Emergency Departments in England and interviews with key informants to examine the hypotheses that underpin GPED. *WP-B; Quantitative Analysis of National Data:* measurement of the effectiveness, costs and consequences of the GPED models identified in WP-A, compared to a no-GPED model, using retrospective analysis of Hospital Episode Statistics (HES) Data. *WP-C; Case Studies*: detailed case studies of different GPED models using a mixture of qualitative and quantitative methods including: non-participant observation of clinical care, semi-structured interviews with staff, patients and carers; workforce surveys with Emergency Department staff and analysis of available local routinely collected hospital data. Prospective case study sites will be identified by completing telephone interviews with sites awarded capital funding by the United Kingdom government to implement GPED initiatives. The study has a strong patient and public involvement group that has contributed to study design and materials, and which will be closely involved in data interpretation and dissemination.

Ethics and dissemination: The study has been approved by the National Health Service East Midlands- Leicester South Research Ethics Committee: 17/EM/0312. The results of the study will be disseminated through peer-reviewed journals, conferences and a planned programme of knowledge mobilisation.

Trial Registration: ISRCTN: 51780222.

For peer review only

Strengths and Limitations of this Study

- We will disseminate a comprehensive assessment of GPED from multiple perspectives to identify models of care which are likely to be most efficient, to maximise clinical and cost-effectiveness, to reduce staff pressure and to improve patient outcome and safety.
- Retrospective analysis of nationally available data, and the associated economic analysis, will be constrained by the quantity and quality of the available information.
- The mixed-methods approach will allow us to elucidate the aims of GPED, the underlying assumptions about how these aims will be achieved, and to assess impacts while considering local context.
- The mixed-methods analysis will also enable the development of recommendations to improve future implementation by identifying challenges and potential solutions.
- GPED models are likely to show significant variation both within and between hospital sites; this may present a challenge to generalisability.

Introduction

Despite many initiatives to reduce demand, pressure from rising attendance rates continues to grow on Emergency Departments in the United Kingdom, with an associated decrease in performance.[1] This leads to Emergency Department crowding, associated with adverse outcomes and increased mortality.[2,3] There is a clear need to find solutions that reduce the burden on Emergency Departments and improve patient experience and safety. In England, the "Keogh Review" of urgent and emergency care aims to reduce pressure on Emergency Departments by treating more patients close to home in primary and community settings.[4] It includes a recommendation that co-located primary care models should be considered in every Emergency Department,[5] however, the optimal model to achieve this has not yet been identified, and evidence for the effectiveness of General Practitioners in the Emergency Department is weak both in the United Kingdom and internationally.[6-9] A recent review of primary care services located with Emergency Departments concluded that there is very little evidence to support this model of care, and that "a robust evaluation… is needed to inform future policy".[10]

Nevertheless, there is an increasing trend to include General Practitioners at the hospital front door. A joint report from four Medical Royal Colleges recommended that every Emergency Department should have a co-located primary care facility.[11] Estimates of the proportion of Emergency Department patients that could be managed by a General Practitioner vary widely between 15% and 40%.[12,13] There are a range of models of integration; most involve General Practitioner services alongside Emergency Department staff, with some operating a separate co-located service as a primary care "filter" in front of the Emergency Department, while others are more integrated with the Emergency Department team. A three-part taxonomy proposed by the Primary Care Foundation [6]

For peer review only

describes three main operational models: a General Practitioner service located alongside or next to the emergency department; General Practitioners working at the front of the department screening attendees and either treating or diverting to other places – effectively acting as a filter; General Practitioner services fully integrated into a joint operation covering the whole range of primary care and emergency services. Recent evidence suggests that some form of co-location exists in 43% of Emergency Departments,[14] however this is likely to have increased rapidly as a result of policy initiatives to increase co-location, including a capital investment of £100 million made by the United Kingdom government in March 2017.[15]

There is also a lack of clarity about the hypothesised mechanism through which locating General Practitioners in Emergency Department will reduce pressure on these departments. Whilst it is has been suggested that implementing a General Practitioner in or alongside the Emergency Department (GPED) may have some benefits for patients, the consequences for the healthcare workforce, both General Practitioners and hospital staff, have not been studied, particularly when there is real uncertainty as to whether GPED reduces Emergency Department attendances,[16] and/or emergency admissions.[17] Some of the apparent impact of GPED on Emergency Departments may simply be re-labelling of the same work, with no real benefits for patients or the National Health Service. Co-located General Practitioner services may further increase demand at hospital sites, transferring the problem of over-crowding from one location to another. In particular, it is not clear what the impact is for General Practitioners, who are already overstretched and in short supply, and GPED may not be the best use of their time and skills.[18] Finally, the cost of implementing and running GPED is a legitimate concern, since employing General Practitioners is likely to be more expensive than employing other types of staff.[19] Although General Practitioners in the Emergency Department may be effective in reducing emergency admissions, they may not be cost effective.[9]

For patients as service users, expectations and regard for their care and experience are important considerations. Research indicates that patients may attend the Emergency Department with non-urgent health problems because of the ease of access or a perceived need for diagnostic tests.[20] Attendances at Emergency Departments are a small proportion of all urgent care consultations when compared to General Practitioner surgeries in the surrounding area, so small shifts in patient behaviour away from General Practice could have major implications for the demand on Emergency Departments.[21]

Effective evaluation of the different models of GPED, including various approaches to patient triage and streaming, and the extent of integration with existing Emergency Department services, is essential to inform service development and meet the urgent heath needs of the population. As a result, this issue will remain highly relevant and important to the future needs of healthcare systems. There is a lack of clarity about the intended benefits and mechanisms of GPED, and little evidence to underpin existing hypotheses.

For peer review only

Research question

What is the impact of General Practitioners working in or alongside the Emergency Department (GPED) on patient care, the primary care and acute hospital team and the wider urgent care system? What is the differential impact of alternative models of GPED?

The study will use a mixed-methods approach, divided into three Work Packages (WPs) (Figure One).

This study is an investigation of complex processes and systems and requires the use of both quantitative and qualitative data. Quantitative methods will be used in WP-B to address research questions about causality, effectiveness, costs and consequences. Qualitative methods will be used in WP-A and WP-C to develop in-depth insight into the main models of GPED care; this will include understanding the aims of introducing GPED and the expected outcomes. These hypotheses will vary according to the context of the service being introduced.

This approach draws upon the strengths of both quantitative and qualitative paradigms. Qualitative data will enable us to describe the current range of models and the mechanisms by which they operate, and to understand the intended aims of the policy. Quantitative data will allow us to test whether the aims have been achieved, whilst the synergies arising from a mixed methods approach to data analysis and interpretation will enable us to explore in depth how and why GPED did or did not work in the way intended in different contexts. This will enable us to make meaningful comparisons across sites, and for different models of GPED.

FIGURE ONE: STUDY FLOW DIAGRAM

For peer review only

Methods and Analysis

See Table One for a summary of research objectives, work packages and methods.

Research Objectives	Work Packages	Methods
1. To map and describe current models of GPED in England	**WP-A**	System leader interviews and documentary analysis
2. To determine the impact of GPED on patient processes and outcomes including overall attendances, attendances in different components of the local urgent care system, waiting times, emergency admissions, re-attendances and mortality	**WP-B**<br><br>**WP-C**	Retrospective analysis of Hospital Episode Statistics data<br>Analysis of local data and<br>non-participant observation
3. To assess the impact of GPED on the case-mix of admitted patients by exploring admission rates, including the number and proportion of short stay and zero day admissions, subject to an examination of coding behaviour by hospital trusts, and any changes that may undermine the reliability of this measure	**WP-B**	Retrospective analysis of Hospital Episode Statistics data
4. To explore the impact of GPED on General Practitioners, including turnover, absence, satisfaction, well-being and attitudes to and scope of practice	**WP-C**	Mixed-methods approach including workforce surveys and interviews
5. To explore the impact of GPED on the working patterns and roles of other health care professionals in the Emergency Department, including training, workload, skill-mix and expertise	**WP-C**	Mixed-methods approach including workforce surveys and interviews
6. To explore the impact of GPED on local urgent care services, on the wider system including primary care (e.g. demand for in-hours and out-of-hours General Practitioner appointments), and on the interface between services including patient flow	**WP-C**	Mixed methods approach using secondary data analysis and qualitative techniques
7. To assess the impact of GPED on patients and carers	**WP-C**	Interviews and non-participant observation
8. To compare resource utilisation and costs of care at Emergency Department sites with and without General Practitioners in or alongside the Emergency Department, and to compare the costs of different service models.	**WP-B**	Economic analysis
9. To prospectively evaluate the current promotion of GPED models of care through collaboration with sites that have bid for capital funding to implement GPED, conducting interviews with identified system leaders and measuring changes in the above parameters over time and as implementation occurs	**WP-C**	Prospective mixed-methods case study approach

For peer review only

TABLE ONE: RESEARCH OBJECTIVES, WORK PACKAGES AND METHODS

WP-A: Mapping and Taxonomy

We will map, describe and classify current models of GPED in all Emergency Departments in England and examine the hypotheses that underpin GPED and its anticipated benefits.

We will work with the Royal College of Emergency Medicine and NHS England to identify, describe and classify current models of General Practitioner working in all Emergency Departments in England, exploring the nature of these co-locations, current service configuration, local funding arrangements and the date of commencement of any service change(s). We will triangulate these sources with Care Quality Commission data, direct enquiry to individual sites and relevant data available from other researchers with an interest in this subject area to understand and classify current models of care, building on the three-part taxonomy proposed by the Primary Care Foundation.[6]

We will rank the identified models of care in order of frequency, and anticipate that two or three distinct model types are likely to emerge which we can then describe and examine in more detail through WP-B and WP-C. Work will also be carried out to establish the hypotheses that underpin GPED. We will approach senior clinicians and managers in selected national organisations including NHS England and the Department of Health, inviting them to participate in a semi-structured interview that will explore their views on GPED and the potential advantages and disadvantages of this service configuration. We will conduct approximately 10-12 interviews that will be recorded digitally, transcribed verbatim and analysed thematically to identify anticipated benefits and impacts of the main GPED models.

WP-B: Quantitative Analysis of National Data

We will measure the impact of the GPED models identified in WP-A, compared to a no-GPED model, using retrospective analysis of Hospital Episode Statistics data. We will adopt a quasi-experimental approach using a repeated interrupted time series design and estimate difference-in-difference regression models with closely matched non-GPED sites as controls. A cost-effectiveness analysis will also be conducted on different GPED models based on their estimated effects alongside resource use.

In this work package we will conduct a quantitative analysis of administrative data to measure the effectiveness, costs and consequences of the most prevalent models of GPED being implemented in hospitals in England.

For peer review only

Methods

We will analyse Emergency Department attendance data, Hospital Episode Statistics inpatient data, and Hospital Episode Statistics Accident and Emergency data for the period 2010-11 to 2015-16. This analysis will be extended to later periods as more data become available during the lifetime of the project. These data will be gathered to address the primary and secondary outcomes of WP-B, which are listed in Table Two.

**Primary Outcome**
Number of Emergency Department attendances
**Secondary Outcomes**
4 hour performance
Unplanned Emergency Department re-attendance within 7 days
Patients leaving the Emergency Department without being seen
Mortality within 28 days of attendance
Emergency hospital admission
Zero day admission (subject to an examination of coding behaviour by hospital Trusts)

TABLE TWO: SUMMARY OF PRIMARY AND SECONDARY OUTCOMES FOR WP-B

National data will be used unless there are specific reasons to exclude individual hospitals, for instance on the grounds of data quality. There is no sampling for this work package, therefore a sample size calculation is not appropriate.

This work package will also include costs and consequences calculations of different GPED models based on their estimated effects on the outcomes listed in Table 2 alongside estimated resource use (e.g. General Practitioner salaries, incremental change in other staffing levels and costs), all derived from routine administrative datasets and local datasets (WP-C) supplemented by information from WP-A. We will use Personal Social Service Research Unit cost estimates supplemented by local cost estimates to value changes in activity and resource inputs. We will use information on the most common funding arrangements for GPED (from WP-A) to differentiate between costs that fall on hospital and primary care budgets, with the objective of identifying genuine changes in resource utilisation rather than cost shifting.

Analysis

We will adopt a quasi-experimental approach, using a combination of interrupted time series and difference-in-difference regression models to identify the causal effect of each GPED model on the primary and secondary WP-B outcomes. We will initially pool all hospitals and GPED types to identify levels and trends before and after implementation, using a multi-level approach. Trends in each hospital and subgroup (based on date of service introduction) will be examined. We will also conduct stratified analysis by GPED model and

For peer review only

by early/late adopter status to determine whether there are differences in effects, and as such which model of GPED is most effective.

WP-C: Case Studies

Detailed mixed-methods hospital case studies will be completed to examine the effect of GPED on staff, patients, flow and resource use within the wider healthcare system. Prospective case study sites will be purposively sampled from telephone interviews with sites awarded capital funding by the United Kingdom government to implement GPED initiatives. Data collection in the case study sites will include:

- Non-participant observation of clinical practice
- Patient and carer interviews
- Emergency Department data, combined with local data sources relating to the wider urgent care system, including primary care data, where available
- Longitudinal interview study and staff surveys administered before and after implementation

In recognition of the limitations of routine data analysis, detailed mixed-methods hospital case studies will be conducted in at least ten sites that are about to implement (at least six sites), or have implemented (at least four sites) a GPED model of care, focussing on the main models identified in WP-A. The prospective sites will be evaluated over time; both before, and 6 and 12 months after, the service change.

Sampling

The case study sites will be selected purposively to ensure a range of characteristics including type of GPED model (2 or 3 options depending on the findings from WP-A), region of England, hospital characteristics (trauma centre, district hospital, volume), population characteristics (affluent, deprived, urban, rural).

Initially, prospective (newly changing) sites will be identified from the bids that have been submitted to the capital fund established in Spring 2017 to support the rapid introduction of new GPED models of care in Emergency Departments in England. Working with NHS England, we will identify a system leader in each of the successfully funded sites and invite them to participate in a telephone interview that will identify the local context, planned model, expected benefits and wider impacts. From this information, sites will be selected based on the criteria described above (six sites). In addition, all those who are interviewed from the successfully funded capital bid sites will be contacted again after 12 months to review progress against the originally stated objectives and assess how successful the implementation of GPED has been.

For peer review only

Established sites that have successfully implemented GPED will be identified during WP-A and WP-B, and on the basis of information provided through professional networks and publications. We anticipate recruiting at least four established sites in WP-C.

Methods

In all ten case study sites a mixture of quantitative and qualitative data will be collected using the following methods.

Quantitative Data

Quantitative data collection in WP-C will include local routine data and the administration of a staff survey.

Local Routine Data

We will obtain routine data about the number and characteristics of patients who have consulted primary and other urgent care services outside the Emergency Department, and any available information about other observable characteristics of the local health economy such as patient outcomes and satisfaction, adverse incidents and reports, to provide contextual data that will help us to interpret findings from the case study sites.

Staff Survey

We will survey staff working in the case study Emergency Department sites to collate their perceptions of GPED using the NoMAD questionnaire (an implementation measure based on Normalization Process Theory (NPT)[22]) alongside standardised and validated measures of work-related experiences and attitudes. The selection of constructs for measurement in this workforce survey will be informed by theoretical models of occupational strain, including the Job Demands-Resources Model,[23] and the Job Demands-Control Model.[24] Specific scales, including measures of job satisfaction and turnover intentions, will be obtained from prior organisational research,[25] and with reference to major data sources (such as the National General Practitioner Worklife Survey) that will enable comparison.[26]

The survey will be administered to all relevant staff in each case site. In the prospective case sites, the survey will be administered at two time points, to see if perceptions of a new service change over time. Descriptive data from the questionnaire will be linked to data collection and analysis of the qualitative data. In keeping with current recommendations, a basic descriptive analysis of the questionnaire data will be produced to provide an overview of the perceptions of staff within each case study site.

Qualitative Data

For peer review only

Qualitative data will be collected to ascertain the views and experiences of GPED from the staff and patients at each case study site. This qualitative data collection will comprise non-participant observation and semi-structured interviews with healthcare professionals, patients and carers at each of the case-study sites.

Non-participant Observation

Non-participant observation will provide a nuanced insight into how the GPED service model is working in practice within the study case sites. The observations will consist of two-hour blocks covering different parts of the day/evening and different activities, e.g. clinical and non-clinical work, triage, informal interactions and clinical consultations. It is considered that a maximum of 12-16 hours of observations over a two-week period within each case site will provide sufficient information. Field notes will document everyday working practices, focusing specifically on the nature of the GPED service, how this is operationalised and the response from patients and clinicians. These data will give greater insight into workplace dynamics, relationships, decision-making and the distribution of tasks and responsibilities.

Semi-structured interviews

Approximately 10-15 staff will be purposively selected (to include General Practitioners, Emergency Department doctors and nurses of different grades) at each of the sites to participate in an interview regarding their experiences of working within their service model. Interviews will follow a topic guide and will also discuss day-to-day practices, involvement in management/oversight of the GPED service and the participant's perceived value of GPED.

These interviews will be conducted longitudinally at prospective case study sites, prior to GPED introduction and 12 months after introduction of the new service model.

We will also collect data on the patient/carer experience of GPED, purposively sampling 10-15 patients (and carers where appropriate) who have used the GPED service to participate in a semi-structured interview. Patients will be selected to obtain maximum spread based on age, gender, and reason for consultation. These interviews will be conducted as soon after attendance as possible, to maximize recall, and will follow a topic guide (Table Three).

In the prospective sites we will also conduct semi-structured interviews with key informants (commissioners and heads of service) before the GPED service goes live to gain insights into the reasons behind the choice of model, the expectations for the service, how staff have reacted to the plans and the preparatory processes that have taken place to implement the new service. Interviews will be repeated approximately 6 and 12 months following full implementation of GPED.

For peer review only

**Prospective case study sites: staff interviews**<br>*Pre-implementation of GPED*<br>• expectations of the new model of care<br>• readiness to employ GPED<br>• information on the appropriateness of the preparations made for the introduction of the new service
**Prospective and established case study sites: staff interviews**<br>*Post-implementation of GPED*<br>Experience of the new service and the impact on:<br>• training/education needs<br>• workload; professional boundaries<br>• job satisfaction/stress<br>• clinical practice and risk management<br>• barriers and facilitators to service introduction<br>• additional topics arising from the local quantitative analysis.
**Prospective and established case study sites: key informants**<br>• advantages and disadvantages of the new model of care<br>• perceived effectiveness of the service model in terms of care provision<br>• impact on staff<br>• barriers and facilitators to successful roll out.
**Patient interviews**<br>• reasons for attending the Emergency Department or GPED<br>• the influence of GPED on their decision to attend<br>• confidence in GPED compared to an Emergency Department clinician<br>• impact of GPED on future Emergency Department and/or General Practitioner attendance.<br><br>We will also ascertain their experiences of GPED in terms of:<br>• quality<br>• advice<br>• referrals and post-discharge care<br>• satisfaction with the service<br>• patients will also be asked to explore which aspects of the service are most important to them, and the barriers/facilitators to service use.

TABLE THREE: KEY POINTS OF TOPIC GUIDES FOR STAFF, PATIENT & KEY INFORMANT INTERVIEWS

For peer review only

Data Analysis

The analysis and interpretation of WP-C will integrate both qualitative and quantitative data and is likely to include the following issues:

- The effect of implementing GPED on patient pathways and flow within the local healthcare system, using non-participant observation and routinely available data;
- The impact of GPED on patients and carers and on healthcare staff using interview data, workforce surveys and routinely available data;
- Barriers and facilitators to the implementation of a GPED model of care, and the development of recommendations to improve future implementation by identifying challenges and potential solutions.

Quantitative Analysis

The analyses of routine quantitative data at the 'site' level will be characterised mainly by descriptive statistics that will complement the qualitative information collected, and which will take due account of any seasonal effects. This approach will enable us to look for potential differences and similarities in views within a case site as well as draw out meaningful comparisons across case sites. Descriptive data from the staff survey will initially inform the purposive selection of participants for qualitative interviews. Further analyses of survey data (e.g., regarding job satisfaction and turnover intentions) will be conducted to examine levels and changes across key outcomes over time, and also indicate variables that may explain variance in these outcomes. While regression analyses will be used for the latter purpose, we do not anticipate high levels of statistical power for these analyses, and we will thus focus on reporting of the direction and magnitude of effect size point estimates and 95% Confidence Intervals.

Qualitative Analysis

All interviews will be audio recorded digitally and transcribed verbatim. The computer package NVIVO will be used to manage the data. Following transcription, the interview material will be organised according to analytical headings using a constant comparison approach. To introduce transparency and a systematic approach we will engage in: detailed familiarisation; identification and indexing of key themes; contextualising these themes in relation to the broader dataset; interpretation, within the context of theoretical themes relevant to the interview material.[27]

Regular meetings will be held to discuss the emergent themes from the fieldwork material and explore the potential to 'test' these in the local quantitative data. The analysis will allow us to gain in-depth insight into the main models of GPED care. This approach will enable us to look for potential differences and similarities in views within a case site as well as draw out meaningful comparisons across case sites and for different models of GPED.

For peer review only

Patient and Public Involvement

A Patient and Public Involvement Group will be engaged throughout all stages and WPs of the study, to ensure the perspectives of patients and carers are considered fully. We will use a variety of methods to work with the group including face to face meetings, e-mail, telephone and video conferencing as appropriate and in keeping with the needs of group members. The Patient and Public Involvement Group will be involved in writing the ethics application and developing research instruments and participant information sheets. For WP-A, the Group will help to write the interview schedules. Data collection plans for WP-B will also be reviewed. The Patient and Public Involvement group will also provide feedback at various stages of data analysis, particularly during WP-C, and will contribute to dissemination plans.

Ethics and dissemination

Routine care is not altered by the study, and it therefore does not raise significant ethical issues. All necessary local research governance approvals will be obtained at hospital sites prior to data collection. WP-A includes interviews with NHS staff, and WP-C includes interviews and observations with NHS staff, patients and carers. Appropriate mechanisms to provide written information and informed consent will be instituted for all NHS staff, patient and carer participants. The study will be conducted in accordance with the International Conference for Harmonisation of Good Clinical Practice guidelines (www.ich.org/). The study is registered on the United Kingdom Clinical Research Network and the International Standard Randomised Controlled Trial Number Registry.

This study will be disseminated through the Knowledge Mobilisation Team at the Centre for Academic Primary Care, University of Bristol. The study results will be presented at national and international conferences and will be published in international peer-reviewed journals. The study commenced in June 2017 and is expected to run until May 2020, with a total duration of 36 months.

Discussion

As the number of hospitals implementing GPED increases rapidly, with several competing models in use, the need for definitive evidence regarding the most efficient model of care, and best use of scarce resources, becomes increasingly urgent. The mixed-methods approach described in this protocol will enable the development of recommendations to improve future implementation by identifying trends, challenges and potential solutions to GPED service change. It is anticipated that the study will generate a comprehensive assessment of GPED from multiple perspectives to identify the models of care that are most likely to be efficient, to maximise clinical and cost-effectiveness, to reduce staff pressure and to improve patient outcome, safety and experience in the United Kingdom.

For peer review only

Authors' contributions: The GPED study team, and the contribution made by each individual, are described in the following statement. The Chief Investigator and study guarantor is Jonathan Benger. The research question was developed and the study was designed by Jonathan Benger, Joy Adamson, Helen Baxter, Karen Bloor, Sean Cowlishaw, Tim Doran, Andrew Gibson, Nils Gutacker, Sarah Purdy, Chris Salisbury and Sarah Voss. The study protocol was developed and refined by Jonathan Benger, Katherine Morton, Sarah Voss, Joy Adamson, Helen Baxter, Karen Bloor, Janet Brandling, Sean Cowlishaw, Tim Doran, Andrew Gibson, Nils Gutacker, Dan Liu, Sarah Purdy, Paul Roy, Chris Salisbury, Arabella Scantlebury, Anu Vaittinen, Rose Watson and Sarah Voss. Financial and contract management is provided by Paul Roy. All authors have reviewed, revised and approved the final manuscript.

Funding statement: This work was supported by the National Institute of Health Research (NIHR) Health services & Delivery Research (HS&DR) Programme, project number 15/145/06.

Disclaimer: The views and opinions expressed therein are those of the authors and do not necessarily reflect those of the Health Services & Delivery Research Programme., NIHR, NHS or the Department of Health.

Competing Interests Statement: Jonathan Benger is seconded part-time to the post of National Clinical Director for Urgent Care at NHS England. All other authors declare no conflict of interest.

Ethics approval: Approval for the study has been obtained from the Health Research Authority (HRA). The protocol was reviewed and received a favourable opinion from the National Health Service (NHS) East Midlands- Leicester South Research Ethics Committee- REC: 17/EM/0312.

Provenance and peer review: Not commissioned; peer reviewed for ethics and funding approval prior to submission.

Open Access Statement: This is an open access article distributed in accordance with the terms of the creative commons attribution (CC BY 4.0) license, which permits others to distribute, remix and adapt and build upon this work for commercial use, provided the original work is properly cited.

For peer review only

References

1. Monitor. A&E delays: why did patients wait longer last winter? Monitor, London: 2015.

2. Collis J. Adverse effects of overcrowding on patient experience and care. *Emerg Nurse* 2010;18(8):34- 39.

3. Richardson DB. Increase in patient mortality at 10 days associated with emergency department overcrowding. *Med J Aust* 2006;184:213-6.

4. NHS England. Transforming urgent and emergency care services in England. Urgent and Emergency Care Review End of Phase 1 Report. NHS England, London: 2013.

5. UEC Review Team and ECIST. Transforming urgent and emergency care services in England. Safer, faster, better: good practice in delivering urgent and emergency care. A guide for local health and social care communities. NHS England, London: 2015.

6. Carson D, Clay H, Stern R. Primary Care and Emergency Departments: Report from the Primary Care Foundation. Primary Care Foundation, London: 2010.

7. Berchet C. OECD Working Paper No. 83. Emergency care services: trends, drivers and interventions to reduce demand. OECD, Paris: 2015.

8. Khangura JK, Flodgren G, Perera R, Rowe BH, Shepperd S. Does employing general practitioners to provide care for patients with non-urgent problems in emergency departments decrease resource use and costs? Cochrane Database of Systematic Reviews 2012, Issue 11. Art. No.: CD002097. DOI: 10.1002/14651858.CD002097.pub3.

9. Purdy S. Avoiding hospital admissions. What does the research evidence say? The King's Fund, London: 2010.

10.Ramlakhan S, Mason S, O'Keeffe C, Ramtahal A, Ablard S. Primary care services located with EDs: a review of effectiveness. *Emerg Med J* 2016. doi:10.1136/emermed-2015-204900.

11.Anonymous. Acute and emergency care: prescribing the remedy. Royal College of Emergency Medicine, London: 2014.

12.Royal College of Emergency Medicine and the Patients Association. Time to act: urgent care and A&E – the patient perspective. RCEM, London: 2015.

13.NHS England. Transforming urgent and emergency care services in England. Urgent and Emergency Care Review End of Phase 1 Report. NHS England, London: 2013.

14.Royal College of Emergency Medicine and Urgent Health UK. The Royal College of Emergency Medicine and Urgent Health UK call for the colocation of urgent care services with A&E departments (press statement, 4th November 2015).

15. Lacobucci, G. All A&Es must have GP led triage. *BMJ* 2017;356:j1270.

For peer review only

16.Salisbury C, Chalder M, Hollinghurst S, et al. The impact of NHS walk-in centres on A&E services. Final report. University of Bristol, Bristol: 2006.

17.O'Dowd, A. GPs in A&E could help tackle unsustainable rise in emergency hospital admissions. *BMJ* 2010;341:c3618.

18.Royal College of General Practitioners. RCGP response to College of Emergency Medicine report. RCGP, London: 2015.

19. Monitor. Walk-in centre review: final report and recommendations. Monitor, London: 2014.

20.Giesen P, Franssen E, Mokkink H, van den Bosch W, van Vugt A, Grol, R. Patients either contacting a general practice cooperative or accident and emergency department out of hours: a comparison. *Emerg Med J* 2006;23(9):731-734.

21.Huibers L, Thijssen W, Koetsenruijter J, Giesen P, Grol R, Wensing M. GP cooperative and emergency department: an exploration of patient flows. *J Eval Clin Pract* 2013;19(2):243-9.

22.Finch TL, Girling M, May CR, Mair FS, Murray E, Treweek S, et al. NoMAD: Implementation measure based on Normalization Process Theory. [Measurement instrument].2015. Available at: http://www.normalizationprocess.org

23.Bakker AB, Demerouti E. The job demands-resources model: State of the art. *Journal of Managerial Psychology* 2007;22:309-328.

24.Karasek Jr, RA. Job demands, job decision latitude, and mental strain: Implications for job redesign. *Administrative Science Quarterly* 1979:285-308.

25.Van Dick R, Christ O, Stellmacher J, Wagner U, Ahlswede O, Grubba C, et al. Should I stay or should I go? Explaining turnover intentions with organizational identification and job satisfaction. *British Journal of Management* 2004;15:351-360.

26.Hann M, McDonald J, Checkland K, Coleman A, Gravelle H, Sibbald B, et al. Seventh national GP worklife survey. University of Manchester; Manchester: 2013.

27. Braun V, Clarke V. Using thematic analysis in psychology. *Qualitative Research in Psychology* 2006;3(2):77-101.

FIGURE ONE: STUDY FLOW DIAGRAM

For peer review only

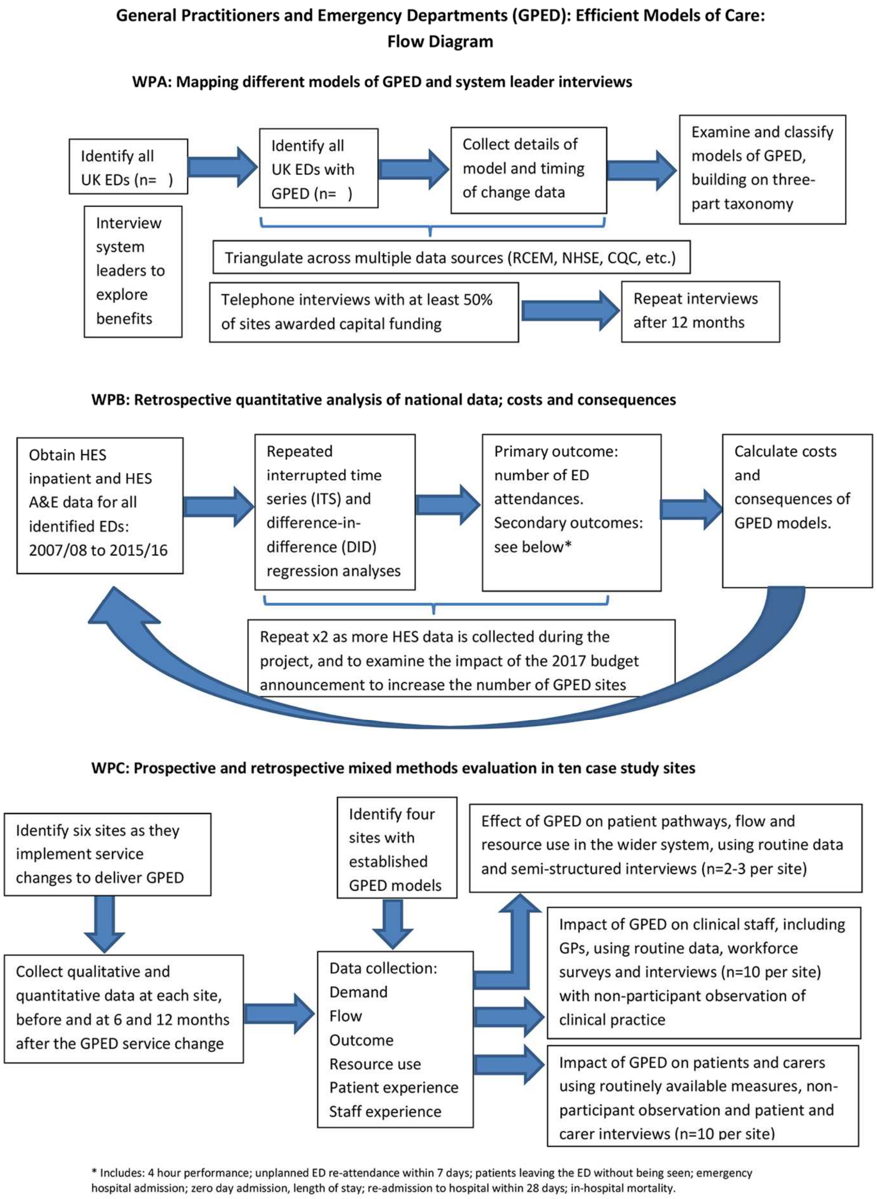

Figure One: Study Flow Diagram

119x165mm (300 x 300 DPI)
