# Supplementary material for: General practitioners and emergency departments (GPED)—efficient models of care: a mixed-methods study protocol
Source: BMJ Open. 2018 Oct 3;8(10):e024012. doi: 10.1136/bmjopen-2018-024012 (PMC6194458; doi:10.1136/bmjopen-2018-024012)
Supplement: Reviewer comments [file bmjopen-2018-024012.reviewer_comments.pdf]

PEER REVIEW HISTORY

BMJ Open publishes all reviews undertaken for accepted manuscripts. Reviewers are asked to complete a checklist review form (**http://bmjopen.bmj.com/site/about/resources/checklist.pdf**) and are provided with free text boxes to elaborate on their assessment. These free text comments are reproduced below.

ARTICLE DETAILS

**TITLE (PROVISIONAL)**	General Practitioners and Emergency Departments (GPED): Efficient Models of Care. A mixed-methods study protocol.
**AUTHORS**	Morton, Katherine; Voss, Sarah; Adamson, Joy; Baxter, Helen; Bloor, Karen; Brandling, Janet; Cowlishaw, Sean; Doran, Tim; Gibson, Andy; Gutacker, Nils; Liu, Dan; Purdy, Sarah; Roy, Paul; Salisbury, Chris; Scantlebury, Arabella; Vaittinen, Anu; Watson, Rose; Benger, Jonathan

This article was not externally reviewed at BMJ Open. Protocols that have been independently assessed prior to submission to BMJ Open are usually fast-tracked to publication on the grounds that further substantial changes will not be possible. This independent assessment will usually be external, independent review for both a substantial grant award from a non-commercial or government funder and ethics approval.
